# Behavioural Drivers of COVID-19 Vaccination and Antiviral Uptake in Australia: A Cross-Sectional Analysis Using the COM-B Framework

**DOI:** 10.3390/vaccines14060495

**Published:** 2026-05-31

**Authors:** Stephen Wiblin, Mohana Kunasekaran, Raina MacIntyre, Holly Seale

**Affiliations:** 1 School of Population Health, University of New South Wales, Sydney, NSW 2000, Australia; h.seale@unsw.edu.au; 2 Kirby Institute, University of New South Wales, Kensington, NSW 2000, Australia; m.kunasekaran@unsw.edu.au (M.K.); r.macintyre@unsw.edu.au (R.M.)

**Keywords:** COVID-19, vaccination, antivirals, COM-B, health behaviour, Australia, LASSO, Cronbach’s alpha

## Abstract

**Objective**: To identify demographic, clinical, and behavioural determinants of COVID-19 vaccination and antiviral uptake in Australia using the Capability, Opportunity, Motivation-Behaviour (COM-B) framework with psychometric validation and LASSO-enhanced variable selection. **Methods**: Cross-sectional analysis of the 2024 KAB BREATHE survey (*n* = 5177) of Australian adults, intentionally enriched for risk-stacked (more than 1 chronic condition). Primary outcomes included 2023/2024 COVID-19 booster receipt, future vaccine intentions, vaccine/antiviral beliefs and antiviral uptake. Predictors included demographics, chronic conditions, and domain-specific leave-one-out (LOO) COM-B scores standardised to mean = 0, SD = 1. COM-B domains were assessed using Cronbach’s alpha. Univariate and multivariable logistic regression models were complemented by LASSO penalised logistic regression with 10-fold cross-validation. **Results**: Among 5177 Australian adults, the mean age was 51.5 years (SD 16.5), 61.4% (3179/5177) were female, and 70.3% (3638/5177) were classified as risk-stacked. Booster uptake declined sharply from 50.8% (2023) to 19.1% (2024). Cronbach’s alpha showed poor internal consistency for Capability (α = 0.006) and Opportunity (α = −0.383) but was acceptable for full Motivation (α = 0.78). In adjusted models, age (aOR 1.02–1.03 per year), medically associated risk factors (aOR 1.66–3.51), and tertiary education (aOR 1.34–1.79) consistently predicted higher uptake and intention. Renting (aOR 0.59–0.78) and current employment (likely inversely associated with age) (aOR 0.73–0.83) were associated with lower uptake across all vaccine outcomes. Adding LOO COM-B scores substantially improved model fit (e.g., 2024 booster AUC 0.73→0.83); Motivation per SD was the strongest predictor (aOR 2.44–4.94 for vaccine outcomes, 1.52–2.49 for antivirals). LASSO models achieved CV-AUCs of 0.78–0.87. Among COVID-positive respondents (*n* = 2576), only 15.2% received antiviral treatment. **Conclusions**: Age, clinical risk, and socioeconomic factors, particularly housing tenure and employment status, are key drivers of COVID-19 preventive behaviours (either positively or negatively). The COM-B framework, when corrected for circular prediction and validated via Cronbach’s alpha and LASSO, provides substantial explanatory value. Targeted interventions should address structural barriers faced by renters and younger, employed individuals while leveraging high motivation among older adults and clinically vulnerable groups. **Implications for Public Health**: These findings support a shift from knowledge-based campaigns towards equity-focused, multi-level public health strategies that address structural barriers to COVID-19 vaccination and antiviral access in Australia.

## 1. Introduction

Understanding the drivers of vaccine hesitancy and treatment uptake is critical to designing effective public health interventions. Knowledge alone is insufficient to drive behaviour; multi-level barriers, including logistical constraints, risk perception, trust deficits, and structural socioeconomic factors, mediate the relationship between awareness and action [1,2]. In Australia, COVID-19 vaccine acceptance has been generally high but with persistent pockets of hesitancy, particularly for booster doses and among some priority groups. Despite broad vaccine availability, booster coverage has declined over time, and COVID-19 continues to contribute substantially to morbidity among older adults and those with chronic conditions [1,2]. The COM-B (Capability, Opportunity, Motivation-Behaviour) framework, developed by Michie et al. (2011) [3], provides a validated, comprehensive structure for mapping behavioural determinants and identifying intervention targets. COM-B posits that behaviour arises from the interaction of three domains: Capability (psychological and physical capacity to engage in behaviour), Opportunity (environmental, social, and structural enablers or barriers), and Motivation (reflective beliefs and intentions, and automatic habits and emotions). The COM-B framework differs from the more vaccination-specific 3C and 5C approaches. Whereas 3C/5C focus on confidence, complacency, constraints/convenience, calculation, and collective responsibility in relation to vaccination, COM-B conceptualises behaviour more broadly as arising from interactions between capability, opportunity, and motivation. This makes COM-B well suited to examining both vaccination and antiviral behaviour within a single framework. Applied to vaccination and antiviral research, COM-B has successfully elucidated why certain interventions succeed or fail [4,5]. However, when behavioural composites are constructed from multiple survey items, there is a risk of circular prediction if outcome-adjacent items are included in predictor scores, which can artificially inflate effect sizes. To minimise this risk, the present study used domain-specific leave-one-out (LOO) COM-B scores and explicitly assessed internal consistency before interpretation [6].

Despite the availability of effective COVID-19 vaccines and oral antiviral treatments, uptake in at-risk populations remains substantially below optimal levels [7]. In Australia, COVID-19 booster coverage has declined precipitously since the acute pandemic phase, with 2024 uptake falling to approximately one-fifth of 2023 levels among surveyed populations. Antiviral prescribing also remains low, with fewer than 20% of eligible COVID-positive individuals receiving treatment in recent cohorts [8]. These trends highlight the need to move beyond descriptive coverage estimates towards a more granular understanding of who is missing out and why.

Australia’s high disease burden among people with multimorbidity, disability, and social disadvantage heightens the urgency of identifying the factors influencing acceptance and uptake of the COVID-19 vaccine [9]. Risk stacking, defined as the accumulation of overlapping clinical (comorbidity, frailty) and social (housing insecurity, language barriers, digital exclusion) vulnerabilities, predicts not only severe infection outcomes but also missed opportunities for vaccination and antiviral access [10,11]. While prior studies have examined vaccine confidence, hesitancy, and uptake in specific populations, fewer have explicitly considered how overlapping clinical and social vulnerabilities interact with a broad behavioural framework such as COM-B, and fewer still have extended that approach to antiviral treatment behaviour. The present study adds to this literature by examining vaccination and antiviral outcomes together in a large national Australian survey using psychometric checks, theory-informed modelling, and LASSO-based variable selection.

Accordingly, this study sought to quantify demographic, clinical, and behavioural determinants of COVID-19 vaccine and antiviral uptake using domain-specific, methodologically corrected COM-B scores with psychometric validation (Cronbach’s alpha); assess variable importance and predictive performance using LASSO-penalised logistic regression; identify risk-stacked subgroups with suboptimal uptake to inform targeted public health strategies; and evaluate the incremental explanatory value of COM-B constructs beyond standard demographic and clinical predictors.

## 2. Methods

### 2.1. Study Design and Population

A cross-sectional analysis of the 2024 wave of the KAB (Knowledge, Attitudes, Behaviour) BREATHE (Behavioural Responses, Engagement, Attitudes, Testing, and Health-seeking Evaluation) survey was undertaken. The BREATHE study is a national, multi-wave online survey of Australian adults aged 18 years and over, administered as part of the NHMRC Centre of Research Excellence (CRE) for mitigating airborne threats to health (Grant Number: GNT1198902). Participants were recruited via an online survey platform using quota-based sampling to achieve coverage across major cities, inner regional areas, and outer regional/remote locations, while intentionally oversampling priority populations including adults aged 70 years and over, people with chronic medical conditions, culturally and linguistically diverse communities, and healthcare or first-responder workers. Survey participation was voluntary, all participants provided informed consent electronically before commencing the questionnaire, and responses were captured through the web-based survey platform rather than hand-delivered instruments or clinic-based recruitment. Quota sampling was selected to improve representation of population groups of public health interest rather than to generate a probability sample. As with all online cross-sectional surveys, selection bias remains possible because participation depended on internet access, willingness to participate, and panel availability. The survey was administered online between 2023 and 2024.

Quota sampling was used to achieve balanced representation across geographic regions (major city, inner regional, outer regional/remote) and to intentionally oversample priority populations: adults aged over 70 years; individuals with chronic medical conditions; culturally and linguistically diverse (CALD) communities; and healthcare and first responder workers. The analytic sample size (*n* = 5177) was determined by quota-based recruitment for the 2024 survey wave rather than by a formal a priori power calculation. Because the survey was administered through an existing online survey and detailed counts at each recruitment step were not recorded, we report analytic denominators for each outcome rather than a full participant flow diagram. The final analytic sample comprised 5177 respondents with valid survey responses. All participants provided informed consent electronically. Ethics approval was obtained from the UNSW Human Research Ethics Committee (approval number HC220737).

### 2.2. Survey Instrument

The KAB BREATHE survey was a purpose-built online instrument developed by the research team. The survey comprised 13 sections with a combination of multiple-choice and open-ended questions, with built-in skip logic to reduce respondent burden. The instrument was reviewed by senior public health officials from New South Wales and Victoria prior to administration. This analysis focuses on data from the sections covering eligibility screening, demographics, and KAB regarding COVID-19 vaccination and antiviral treatments (Appendix A).

### 2.3. Predictor Variables

#### 2.3.1. Demographic and Clinical Covariates

Core adjustment variables included: age (continuous, years); gender (binary: female vs. male/other); country of birth (binary: overseas vs. Australia); language at home (binary: non-English vs. English only); education (binary: tertiary [Bachelor/postgraduate] vs. non-tertiary); employment (binary: currently employed [full-time/part-time/self-employed] vs. other); healthcare/first responder status (binary: yes vs. no); housing tenure (binary: renting vs. own outright/mortgage); area of residence (binary: rural/remote vs. major city/inner regional); any chronic condition (binary: self-reported diagnosis of one or more chronic diseases); any medical risk factor (binary: self-reported presence of one or more specific COVID-19 risk factors, including disability with multiple conditions, neurological conditions, chronic respiratory conditions, obesity or diabetes requiring medication, heart failure, coronary artery disease, kidney failure or cirrhosis, and living remotely with reduced access to higher-level healthcare); and risk stacked (binary: age over 70 years, or any chronic condition, or any medical risk factor in combination).

#### 2.3.2. COM-B Scores: Domain-Specific Leave-One-Out (LOO) Methodology

To mitigate circular prediction, domain-specific COM-B scores were constructed using a leave-one-out approach. For each outcome variable, any thematically related COM-B item with a Pearson correlation |r| > 0.40 with that outcome was excluded from the composite score before regression.

Motivation items (raw pool) comprised: total COVID-19 vaccine doses received (Q69, continuous, min-max scaled 0–1); vaccine belief and intention scales (Q74, Q77, Q78, Q79, Q80, Q89, scaled 0–1); and sociodemographic indicators (age, language at home, education level, area of residence, scaled 0–1). Opportunity items (raw pool) comprised: any chronic condition (Q23); any Q32 risk factor; paid out-of-pocket for healthcare (Q36); antiviral intentions (Q85, Q86); female gender; and born overseas.

For vaccine intentions and beliefs (Q74, Q77, Q78, Q79, Q80, Q89), the Motivation score excluded all other vaccine attitude items, retaining only Q69, age, language, education, and area of residence. For antiviral outcomes, the Opportunity score excluded self-referential items (e.g., Q85 was excluded when predicting Q85). For each respondent and outcome, the LOO Motivation score was the mean of non-excluded items (requiring >50% non-missing; otherwise set to missing). All final COM-B scores were standardised to mean = 0, SD = 1 before regression.

### 2.4. Psychometric Assessment: Cronbach’s Alpha

Cronbach’s alpha was calculated to assess the internal consistency of the three COM-B domains. Alpha values > 0.70 indicate acceptable internal consistency; values < 0.60 suggest the items do not form a unidimensional scale [12].

### 2.5. Statistical Analysis

All analyses were conducted using Python 3.10. A *p*-value < 0.05 was considered statistically significant; no formal correction for multiple testing was applied, given the exploratory nature of the analysis.

Descriptive statistics included frequencies and percentages for categorical variables and means and standard deviations for continuous variables. All core demographic and clinical covariates were retained in multivariable models a priori on theoretical and public health grounds rather than being selected solely on the basis of univariate *p*-values. The purpose of this modelling structure was to assess the incremental explanatory value of behavioural constructs beyond standard demographic and clinical factors. An intercept-only model was not presented because it was not substantively informative for the study objectives. Complete-case analysis was used throughout. Multiple imputation was not implemented in this survey wave because the present analysis was designed as an initial cross-sectional investigation focused on internally comparable complete-case models across multiple outcomes. The denominator for each analysis is therefore reported explicitly, and differing sample sizes reflect item completion and eligibility. Univariate logistic regression was used to assess unadjusted associations between each outcome and each predictor. Two hierarchical multivariable logistic regression models were fitted for each outcome: Model 2 (Extended Demographics), adjusted for the 11 core demographic and clinical covariates; and Model 3 (Full LOO COM-B), which added the standardised LOO Motivation and Opportunity scores to Model 2. For each model, adjusted odds ratios (aORs), 95% confidence intervals (CIs), *p*-values, and model fit statistics (Akaike Information Criterion, McFadden’s pseudo-R^2^, and area under the receiver operating characteristic curve [AUC]—Table S4 in Supplementary Material) were reported. Complete-case analysis was used throughout.

LASSO (L1-penalised) logistic regression with 10-fold cross-validation was performed as a supplementary analysis to identify the most influential predictors from a larger set of demographic, health, and raw COM-B items, with LOO outcome-exclusion rules applied to avoid information leakage. All predictors were z-standardised before fitting. The penalty parameter (λ) was selected to maximise mean AUC across folds.

Multiple imputation was not implemented in this wave and remains planned for future analyses. Capability scores were excluded from final multivariable models because they are only assessable among COVID-positive respondents (*n* = 2576), substantially reducing sample size for whole-population outcomes.

## 3. Results

### 3.1. Sample Characteristics

The analytic sample comprised 5177 Australian adults. The mean age was 51.5 years (SD 16.5), and 61.4% (3179/5177) were female. Most participants were born in Australia (76.9%, 3980/5177), and 11.6% (602/5177) spoke a language other than English at home. Educational attainment was high, with 41.2% (2136/5177) holding a Bachelor’s degree or higher. Chronic disease burden was substantial, with 65.2% (3373/5177) reporting at least one chronic condition. Over one-quarter had at least one Q32 medical risk factor (26.3%, 1360/5177), and 70.3% (3638/5177) met the study definition of being risk-stacked. Hypertension (21.9%), asthma or chronic respiratory disease (20.3%), diabetes (11.4%), and cancer (8.9%) were the most frequently reported conditions (see Supplementary Table S1). Applying the composite “risk stacked” definition, 70.3% of respondents were classified as at higher risk, confirming the intentional enrichment strategy. Full sample characteristics are presented in Table 1.

### 3.2. Psychometric Assessment of COM-B Domains

Cronbach’s alpha for the SAP-defined COM-B domains showed poor internal consistency for Capability (α = 0.006) and Opportunity (α = −0.383), with all or most items displaying low item-total correlations (ITC < 0.20), indicating multidimensionality. The full Motivation scale (11 items) demonstrated acceptable internal consistency (α = 0.78), while the vaccine-specific LOO Motivation composite (6 items) had poor internal consistency (α = 0.14), consistent with its design as a heterogeneous propensity index. Results are presented in Table 2.

### 3.3. Outcome Distributions

The 2023 booster had been received by 50.8% of respondents, which fell sharply to 19.1% for the 2024 booster (a 62% relative decline). Approximately 60% of respondents intended to receive a new or annual booster (Q74: 60.5%; Q77: 60.2%). Mean total COVID-19 vaccine doses received was 3.9 (SD 1.8). Most respondents agreed that vaccines reduce COVID-19 risk (75.6%; Q78) and reduce COVID-19 severity (79.5%; Q80). However, only 27.7% agreed that vaccines prevent COVID-19 infection (Q79), reflecting widespread awareness of the limitations of vaccines against infection with newer variants. Two-thirds (66.8%) of respondents believed boosters are important (Q89).

Among 2576 COVID-positive respondents, only 15.2% (*n* = 391) received antiviral treatment (Q33). The primary barriers to antiviral receipt were: did not talk to a doctor (57.0%), doctor did not recommend antivirals (23.7%) and did not know antivirals existed (12.8%). Despite low uptake, willingness to take antivirals was moderate to high: 64.9% were somewhat or extremely likely to take antivirals if offered (Q35); 68.3% were likely or very likely to seek antivirals from a GP if eligible (Q85); and 65.8% were likely or extremely likely to take antivirals if eligible (Q86). Outcome distributions are summarised in Table 3.

### 3.4. Univariate Predictors

Univariate logistic regression identified several predictors that were consistently associated with vaccination and antiviral outcomes. For brevity, the full univariate results, including odds ratios and 95% confidence intervals for all predictors and outcomes, are provided in Supplementary Table S2. The main text summarises the direction and consistency of crude associations, while Table 4 and Table 5 present the adjusted findings. Consistent positive predictors (higher uptake/intention) included age (OR 1.02–1.04 per year), chronic conditions (OR 1.5–4.2), medical risk factors (OR 1.75–4.14), risk stacked (OR 1.93–4.06), and tertiary education (OR 1.26–1.67). Consistent negative predictors (lower uptake) included female gender (OR 0.48–0.79), currently employed (OR 0.47–0.60), and renting (OR 0.44–0.71). COM-B Motivation (per SD) showed strong associations (OR 1.53–2.87) across all outcomes, with particularly strong effects for belief outcomes (OR 2.64 for Q79—belief that COVID-19 vaccines prevent infection).

### 3.5. Multivariable Predictors: (Model 2: Extended Demographics)

After adjusting for 11 core demographic and clinical covariates, several predictors remained robustly significant. Key results are presented in Table 4.

### 3.6. Model 3: Impact of LOO COM-B Scores

Adding standardised LOO Motivation and Opportunity scores to the extended demographic substantially improved model fit and discrimination across all outcomes. Age was approximately normally distributed in the analytic sample and is therefore presented as mean (SD); Prior vaccine doses were also indicated as mean (SD) the main remaining variables were binary or categorical and are presented as counts and percentages. The COM-B Motivation score (per SD) was the dominant predictor across all vaccine outcomes, with the largest effect observed for the 2024 booster (aOR 4.94, 95% CI 4.28–5.71, *p* < 0.001). For antiviral uptake, Motivation per SD yielded aOR 1.52 (95% CI 1.31–1.77, *p* < 0.001). The COM-B Opportunity score showed modest, outcome-specific effects. Model fit improvements and COM-B effect sizes are presented in Table 5.

### 3.7. Supplementary LASSO Results

LASSO models achieved comparable or slightly better predictive accuracy than standard logistic regression for most outcomes, with AUCs ranging from 0.770 (antiviral willingness) to 0.871 (boosters important). Across outcomes, LASSO consistently selected a core set of influential predictors including: vaccine attitude items Q78 (vaccines reduce risk) and Q80 (vaccines reduce severity) which had the largest coefficients; age, renting, and Q69 (doses received) were frequently selected with significant coefficients; and many comorbidity variables (cancer, heart disease) were shrunk to zero, suggesting limited direct predictive power after accounting for other factors. The convergence between prespecified logistic models and LASSO in identifying age, medical risk factors (Q32), renting, employment, and prior vaccination history as dominant predictors strengthens confidence in these as core markers of inequity and engagement. Supplementary LASSO results are available in Supplementary Table S3.

## 4. Discussion

This large, cross-sectional analysis of 5177 Australian adults intentionally enriched for at-risk populations provides robust, psychometrically validated evidence of the demographic, clinical, and behavioural drivers of COVID-19 vaccine and antiviral uptake. Five key findings emerge from this analysis, each with distinct implications for public health theory and practice.

### 4.1. Psychometric Limitations of COM-B Constructs

Psychometric assessment showed that the Capability and Opportunity domains had very poor internal consistency, whereas the full Motivation domain performed acceptably. These findings suggest that, in this survey, Capability and Opportunity function better as broad behavioural indicator sets than as coherent unidimensional scales. The low alpha for the vaccine-specific LOO Motivation composite reflects a methodological trade-off: removing outcome-adjacent items reduces circular prediction but also weakens scale coherence [13]. These findings also align with broader reflections that COM-B is most useful as a flexible heuristic framework for understanding and designing behaviour change interventions, rather than as a fixed psychometric scale set [6].

### 4.2. Age and Clinical Vulnerability as Drivers of Uptake

Age and clinical vulnerability (medical risk factors, chronic disease, risk stacking) are the most consistent and clinically meaningful predictors of uptake and intention, with adjusted odds ratios typically 1.5–3.5. This aligns with national and international data showing higher vaccine acceptance among older adults and those with comorbidities [14,15,16]. The increasing effect size of medical risk factors from 2023 (aOR 1.66) to 2024 (aOR 1.98) for booster uptake suggests that as broader population engagement eroded, uptake became increasingly concentrated among the most clinically vulnerable a pattern consistent with the “risk-based” vaccination behaviour observed in other contexts [17].

This trajectory has important equity implications. If booster uptake becomes increasingly stratified by clinical risk, the population-level benefits of vaccination may become concentrated among those who are already most engaged with the healthcare system, while those who are clinically vulnerable but less healthcare-engaged including younger adults with chronic conditions, people with disabilities, and older adults who are socioeconomically disadvantaged may be left behind. This concern is supported by the finding that risk stacking (aOR 1.74–2.15) was a significant predictor of uptake even after adjustment for individual clinical conditions, suggesting that the cumulative burden of overlapping vulnerabilities, rather than any single condition, is the key driver of engagement. Public health programs should therefore move beyond condition-specific targeting to adopt a holistic, risk-stacking lens that identifies individuals with multiple overlapping vulnerabilities.

The finding that female gender was consistently associated with lower vaccination uptake (aOR 0.63–0.74) across all outcomes is notable and somewhat counterintuitive given that women generally have higher rates of healthcare engagement than men. This finding may reflect the disproportionate burden of caregiving responsibilities on women, which can create logistical barriers to vaccination, or it may reflect the higher rates of vaccine hesitancy among women documented in previous Australian studies [14,15]. Targeted messaging that addresses the specific concerns of women may be warranted. These findings are consistent with prior studies showing higher COVID-19 vaccine uptake among older adults and people with comorbidities.

### 4.3. Structural Barriers: Housing Tenure and Employment

Socioeconomic and structural factors particularly housing tenure and employment status are powerful, independent predictors of lower uptake. Renters had 22–40% lower odds (aOR 0.59–0.78) and currently employed individuals had 17–32% lower odds (aOR 0.68–0.83) across all outcomes, even after full adjustment for clinical and demographic covariates. These findings suggest that housing insecurity and work-related time constraints represent critical, modifiable barriers not captured by traditional risk stratification [18,19,20,21].

The consistent LASSO selection of renting with negative coefficients across all vaccine outcomes, alongside its strong effects in prespecified models, provides convergent evidence from two independent analytical approaches that housing tenure is a critical structural determinant of COVID-19 preventive behaviour. This finding is consistent with the broader literature linking housing insecurity to reduced healthcare access and engagement [18,19]. The mechanisms are likely multifactorial: renters may face greater residential mobility, reducing continuity of care and the opportunity for GP-initiated vaccination; they may experience greater financial stress, reducing their capacity to prioritise preventative health; and they may be more likely to live in overcrowded conditions that increase exposure risk while simultaneously reducing the perceived personal benefit of vaccination.

The employment paradox whereby currently employed individuals have lower vaccination uptake despite generally higher socioeconomic status is a particularly important finding for program design. This finding challenges the conventional assumption that socioeconomic advantage is uniformly associated with higher health engagement and suggests that the logistical barriers of employment (time off work, managing side effects) may outweigh the motivational advantages of higher income and education in the context of booster vaccination. This is consistent with the concept of “structural opportunity” in the COM-B framework: even highly motivated individuals may fail to act if the structural conditions do not support the behaviour [3]. Practical solutions include workplace vaccination programs, extended clinic hours, and pharmacy-based vaccination that do not require time off work.

Beyond these structural constraints, perceptions of risk exposure, overall health status, and capacity to manage competing demands likely shape how renters and working-age adults interpret the need for COVID-19 prevention. Many people who perceive themselves as generally healthy, or who normalise frequent exposure in crowded workplaces and shared housing environments, may downplay their personal risk despite objective vulnerability, a pattern consistently reported in COVID-19 vaccine studies where lower perceived susceptibility and severity predict reduced uptake even among clinically at-risk groups [22,23]. Emerging evidence also suggests that psychological distress, stigma, and competing day-to-day stressors can weaken the link between self-efficacy and vaccination intentions, particularly when access barriers are high [24].

Interventions that explicitly acknowledge these lived pressures, reframe antivirals and boosters as tools to maintain work and caregiving capacity (rather than only as responses to severe illness),and provide easy “on the way” access such as extended clinic hours, walk-in services near public transport hubs, pharmacy-based vaccination and antiviral pathways, and bulk-billed telehealth consultations may therefore be particularly important for structurally constrained groups [25,26].

Messaging that emphasises the personal and economic benefits of avoiding even mild-to-moderate illness (for example, avoiding lost workdays, maintaining caregiving responsibilities) may resonate more effectively with this demographic than traditional risk-based appeals focused on hospitalisation prevention alone. Similar associations between structural constraints, healthcare access, and lower uptake have been reported in other COVID-19 vaccination studies.

### 4.4. COM-B Motivation as a Behavioural Marker

The COM-B framework provides substantial explanatory value beyond demographics alone. Adding LOO COM-B scores improved discrimination markedly (e.g., 2024 booster AUC 0.73→0.83). Motivation was the strongest predictor (aOR 1.5–4.9), confirming that accumulated pro-vaccine orientation shaped by experience, trust, and structural context drives behaviour even after rigorous correction for circular prediction. This finding is consistent with the broader literature demonstrating the importance of motivational factors in vaccination behaviour [3,4,5].

The very large aOR for the belief that vaccines prevent infection (Q79; aOR 28.62) warrants careful interpretation. This extreme effect size reflects the residual conceptual overlap between the Motivation composite and the Q79 belief item: even after LOO exclusion, the remaining Motivation items (prior doses, age, education, language) are strongly correlated with vaccine beliefs, creating a situation where the Motivation score is effectively a proxy for the entire pro-vaccine belief system. This is not a failure of the LOO methodology per se, but rather a reflection of the deeply interconnected nature of vaccine attitudes and behaviours such that individuals who have received multiple prior doses, who are older, and who are more educated are simultaneously more likely to hold pro-vaccine beliefs, to have high Motivation scores, and to receive boosters. Disentangling these constructs requires longitudinal data with prospective measurement of attitudes before vaccination decisions are made.

The modest improvement in model fit for antiviral outcomes (ΔAUC +0.013 for Q33) compared to vaccine outcomes (ΔAUC +0.100 for Q66) is also informative. This difference suggests that the COM-B Motivation composite which is anchored primarily in prior vaccination history and vaccine attitudes is a less effective predictor of antiviral behaviour than of vaccination behaviour. This is consistent with the finding that the primary barrier to antiviral uptake was not attitudinal (low willingness) but structural (failure to consult a doctor). Future COM-B instruments for antiviral research should therefore include items specifically targeting antiviral knowledge, perceived eligibility, and healthcare access barriers. This pattern is also consistent with literature showing that motivational and attitudinal orientation remain strong correlates of vaccine behaviour even after demographic adjustment.

### 4.5. Decline in Booster Uptake and Its Public Health Implications

The 62% relative decline in booster uptake from 2023 (50.8%) to 2024 (19.1%) is a striking finding with significant public health implications. This decline is consistent with national trends and reflects a combination of pandemic fatigue, changing risk perception, and reduced public health messaging intensity [17,27]. LASSO analysis revealed that prior doses received (Q69) was consistently selected as a top predictor, confirming that vaccination history is a powerful marker of future behaviour likely reflecting both trust accumulated through positive experiences and stable pro-vaccine orientation [14].

This trajectory is concerning in the context of Australia’s ageing population and the ongoing burden of COVID-19 in high-risk groups. The 2024 booster uptake of 19.1% in this intentionally enriched, high-risk sample is likely an overestimate of uptake in the general population, given the overrepresentation of individuals with chronic conditions and medical risk factors. If booster uptake continues to decline at this rate, the population-level protection conferred by vaccination will erode rapidly, particularly among older adults and those with comorbidities who are most at risk of severe outcomes.

The finding that approximately 60% of respondents still intend to receive a future booster (Q74: 60.5%; Q77: 60.2%), despite only 19.1% having received the 2024 booster, suggests a significant intention–behaviour gap. This gap where individuals express positive intentions but fail to act is a well-documented phenomenon in health behaviour research and is typically explained by the absence of specific plans, logistical barriers, or competing priorities [3]. Targeted interventions that convert intention into action such as reminder systems, pre-booked appointments, and proactive outreach from GP are likely to be more effective than campaigns aimed at building general vaccine confidence, given that much of the population already holds positive vaccine beliefs.

### 4.6. Critically Low Antiviral Uptake

Antiviral uptake was low, with only 15.2% of COVID-positive respondents reporting treatment receipt, despite substantially higher stated willingness to seek or take treatment if eligible. This pattern suggests that the main obstacle is not strong resistance to treatment but failure to connect eligible patients with timely care during the short antiviral treatment window [28]. Potential policy responses in Australia include rapid-access telehealth assessment, pharmacy-linked referral or prescribing pathways where permitted, clearer public messaging on eligibility and treatment timing, and proactive clinician prompts for higher-risk patients.

### 4.7. Strengths and Limitations

Strengths of this study include a large, intentionally diverse sample; comprehensive outcome assessment spanning vaccination, antiviral uptake, intentions, and beliefs; methodologically corrected COM-B scores with psychometric validation; LASSO-enhanced variable selection; and transparent reporting of convergent evidence across modelling approaches. Limitations are substantial. As a cross-sectional survey, the study cannot establish temporality or causality between attitudes and behaviours. Online quota-based recruitment may also have introduced selection bias, and self-reported vaccination and antiviral use are subject to recall and social desirability bias. Residual confounding may therefore remain despite multivariable adjustment. The cross-sectional design precludes causal inference or assessment of temporal precedence. The poor internal consistency of Capability and Opportunity domains limits their use as validated scales. Residual COM-B overlap means that large odds ratios for attitudinal outcomes reflect residual coupling rather than discrete causal effects. Self-report bias is a concern, as vaccination status, antiviral use, and chronic conditions were self-reported without clinical validation. The intentional oversampling of risk-stacked and CALD populations limits direct generalisability to the overall Australian population without weighting. No multiple testing correction was applied, elevating the risk of Type I error across the large number of hypothesis tests conducted. Finally, LASSO was used for variable importance assessment rather than primary inference.

## 5. Conclusions

This study demonstrates that COVID-19 vaccination and antiviral behaviours among at-risk Australian adults are shaped by the interplay of clinical vulnerability, motivational orientation, and structural socioeconomic conditions, rather than by knowledge alone. Applying a refined COM-B approach highlighted both the promise and the limitations of using behavioural theory as a measurement tool, underscoring that multi-item composites must be interpreted cautiously when they aggregate diverse constructs.

The findings point to a small number of priority groups and mechanisms that cut across outcomes: people experiencing housing insecurity, those juggling paid work and caregiving responsibilities, women, and individuals who do not routinely engage with primary care during acute illness. Addressing the needs of these groups will require equity-focused strategies that reduce logistical and financial barriers, embed vaccination and antiviral access into the settings where people live and work, and leverage trusted relationships with general practitioners, pharmacists, and other frontline providers.

More broadly, the results support a shift from generic, knowledge-based campaigns towards integrated, system-level interventions that link risk communication, service design, and policy levers such as funding models, outreach programs, and scope-of-practice reforms. Future research may use longitudinal, linked designs to track how evolving risk perception, trust, and structural conditions influence booster and antiviral decisions over time and to test whether targeted COM-B-informed interventions can narrow persistent gaps for risk-stacked populations. These findings suggest several practical interventions for the Australian context, including extended-hours and workplace vaccination delivery, pharmacy-based vaccination and referral pathways, proactive GP reminders for eligible patients, and rapid telehealth access to antiviral assessment. Such approaches may help reduce the structural barriers identified for renters, working-age adults, and other groups who appear willing to act but face practical barriers to uptake.

## Figures and Tables

**Table 1:** Sample characteristics (*n* = 5177).

Variable	Category/Value	n (%)
Age (years)	Mean (SD)	51.5 (16.5)
Gender	Female	3179 (61.4%)
	Male	1966 (38.0%)
Born overseas	Yes	1197 (23.1%)
Non-English at home	Yes	602 (11.6%)
Tertiary education	Yes	2136 (41.2%)
Currently employed	Yes	3113 (60.1%)
Renting	Yes	1998 (38.6%)
Rural/remote area	Yes	426 (8.2%)
Any chronic condition	Yes	3373 (65.2%)
Any medical risk factor *	Yes	1360 (26.3%)
Risk stacked	Yes	3638 (70.3%)

* Note. Q32 risk factors include: disability with multiple conditions and/or frailty, neurological conditions, chronic respiratory conditions (COPD, moderate or severe asthma), obesity or diabetes requiring medication, heart failure, coronary artery disease, cardiomyopathies, kidney failure or cirrhosis, and living remotely with reduced access to higher-level healthcare.

**Table 2:** Internal consistency of COM-B domains (Cronbach’s alpha).

Domain	N (Items)	Cronbach’s α	Interpretation	Key Low-ITC Items (<0.20)
Capability	5	0.006	Poor	Q34 (courses), Q43 (severity), Q44 (received)
Opportunity	7	−0.383	Poor	All items had ITC < 0.20
Motivation (Full)	11	0.779	Acceptable	Q69 (doses), Q150 (language), Q152 (edu), Q163 (area)
Motivation (LOO—Vaccine)	6	0.136	Poor	All items had ITC < 0.20

Note. ITC = item-total correlation. Alpha values > 0.70 indicate acceptable internal consistency; values < 0.60 suggest items do not form a unidimensional scale [12].

**Table 3:** Key outcome distributions.

Outcome	Measure	n	N	n/N (%) or Mean (SD)
Vaccine Uptake				
2023 booster received (Q63)	Yes	2538	4996	50.8%
2024 booster received (Q66)	Yes	968	5066	19.1%
Vaccine Intentions				
Intend to get new booster (Q74)	Yes	2500	4131	60.5%
Support annual booster (Q77)	Yes	2531	4204	60.2%
Mean total doses received (Q69)	—			3.9 (SD 1.8)
Vaccine Beliefs				
Vaccines reduce COVID-19 risk (Q78)	Agree/strongly agree	3913	5177	75.6%
Vaccines prevent COVID-19 infection (Q79)	Agree/strongly agree	1434	5177	27.7%
Vaccines reduce COVID-19 severity (Q80)	Agree/strongly agree	4117	5177	79.5%
Boosters are important (Q89)	Yes	2800	4193	66.8%
Antiviral Uptake and Intentions				
Received antiviral treatment (Q33)	Yes	391	2576	15.2%
Willing to take antivirals if offered (Q35)	Somewhat/extremely likely	3336	5141	64.9%
Would seek antivirals from GP if eligible (Q85)	Likely/very likely	3535	5177	68.3%
Would take antivirals if eligible (Q86)	Likely/extremely likely	3405	5177	65.8%

**Table 4:** Adjusted odds ratios from multivariable logistic regression for selected outcomes.

Predictor	2024 Booster (Q66) aOR (95% CI)	New Booster Intention (Q74) aOR (95% CI)	Antiviral Uptake (Q33) aOR (95% CI)
Age (per year)	1.02 (1.02–1.03) ***	1.02 (1.02–1.03) ***	1.02 (1.01–1.03) ***
Female gender	0.63 (0.54–0.74) ***	0.74 (0.64–0.85) ***	0.66 (0.52–0.84) ***
Tertiary education	1.42 (1.21–1.66) ***	1.63 (1.41–1.88) ***	1.34 (1.04–1.73) *
Currently employed	0.73 (0.61–0.87) ***	0.71 (0.61–0.83) ***	0.75 (0.56–0.99) *
Renting	0.59 (0.49–0.70) ***	0.72 (0.62–0.83) ***	0.67 (0.51–0.89) **
Any medical risk factor	1.98 (1.68–2.34) ***	1.56 (1.32–1.85) ***	3.51 (2.72–4.53) ***
Risk stacked	1.74 (1.24–2.45) **	—	2.15 (1.24–3.75) **

Note. aOR = adjusted odds ratio; CI = confidence interval. * *p* < 0.05; ** *p* < 0.01; *** *p* < 0.001. Dashes indicate predictor not significant in that model. Sample sizes: Q33 n = 2562 (COVID-positive respondents only); Q66 n = 5066; Q74 n = 4131.

**Table 5:** Model fit improvements and COM-B Motivation effect sizes (Model 2 vs. Model 3).

Outcome	Model 2 AUC	Model 3 AUC	ΔAUC	Model 2 Pseudo-R^2^	Model 3 Pseudo-R^2^	COM-B Motivation aOR (95% CI)
2024 booster (Q66)	0.728	0.828	+0.100	0.104	0.233	4.94 (4.28–5.71) ***
2023 booster (Q63)	—	—	—	—	—	2.44 (2.20–2.71) ***
New booster intention (Q74)	—	—	—	—	—	2.47 (2.20–2.78) ***
Belief vaccines prevent infection (Q79)	0.637	0.896	+0.259	0.047	0.392	28.62 (23.38–35.03) ***^†^
Antiviral uptake (Q33)	0.759	0.772	+0.013	—	—	1.52 (1.31–1.77) ***

Note. AUC = area under the receiver operating characteristic curve; Pseudo-R^2^ = McFadden’s pseudo-R^2^. *** *p* < 0.001. ^†^ The extremely large aOR for Q79 reflects residual conceptual overlap between the Motivation composite and vaccine belief items and should be interpreted as a marker of attitudinal positioning rather than a discrete causal effect.

## Data Availability

Informed consent was obtained from all subjects involved in the study. The data presented in this study are available on request from the corresponding author.

## Supplementary Materials

The following supporting information can be downloaded at https://www.mdpi.com/article/10.3390/vaccines14060495/s1, Table S1: LASSO Standardised Coefficients—Selected Predictors by Outcome; Table S2: Univariate Logistic Regression Results; Table S3: LASSO Penalised Logistic Regression—10-fold Cross-Validation; Table S4: Model Fit Statistics.

## Appendix A. Survey Instrument

**Outcome Domain**	**Specific Outcome/Construct**	**Survey Item (Q No.)**	**Coding Notes**
Vaccine uptake	2023 COVID-19 booster received	Q63	Binary: yes/no
Vaccine uptake	2024 COVID-19 booster received	Q66	Binary: yes/no
Vaccine intentions	Intend to receive anew/next COVID-19 booster	Q74	Binary: yes/no; “unsure” excluded
Vaccine intentions	Support for yearly COVID-19 boosters	Q77	Binary: yes/no; “unsure” excluded
Vaccine beliefs	Belief that vaccines reduce COVID-19 risk	Q78	Binary: agree/strongly agree vs. disagree/strongly disagree
Vaccine beliefs	Belief that vaccines prevent COVID-19 infection	Q79	Binary: agree/strongly agree vs. disagree/strongly disagree
Vaccine beliefs	Belief that vaccines reduce COVID-19 severity	Q80	Binary: agree/strongly agree vs. disagree/strongly disagree
Vaccine beliefs	Belief that boosters are important	Q89	Binary: yes vs. no; “unsure” excluded where applicable
Antiviral uptake	Receipt of oral antiviral treatment among COVID-positive respondents	Q33	Binary: yes/no
Antiviral intentions	Willingness to take antivirals if offered	Q35	Binary: likely/very likely vs. unlikely/not at all likely
Antiviral intentions	Likely to seek antivirals from a GP if eligible	Q85	Binary: likely/very likely vs. unlikely/not at all likely
Antiviral intentions	Likely to take antivirals if eligible	Q86	Binary: likely/very likely vs. unlikely/not at all likely

## References

[1] Dubé E., Laberge C., Guay M., Bramadat P., Roy R., Bettinger J. (2013). Vaccine hesitancy: An overview. Hum. Vaccines Immunother..

[2] MacDonald N.E., SAGE Working Group on Vaccine Hesitancy (2015). Vaccine hesitancy: Definition, scope and determinants. Vaccine.

[3] Michie S., van Stralen M.M., West R. (2011). The behaviour change wheel: A new method for characterising and designing behaviour change interventions. Implement. Sci..

[4] Patterson L., Berry E., Parsons C., Clarke B., Little A., Beggs J., Chuter A., Jackson T., Hsia Y., McGrath H. (2023). Using the COM-B framework to elucidate facilitators and barriers to COVID-19 vaccine uptake in pregnant women: A qualitative study. BMC Pregnancy Childbirth.

[5] Smith L.E., Sim J., Cutts M., Dasch A., Amlôt R., Sevdalis N., Rubin G.J., Lambert H. (2023). Psychosocial factors affecting COVID-19 vaccine uptake in the UK: A prospective cohort study (CoVAccS—Wave 3). Vaccine X.

[6] Whittal A., Atkins L., Herber O.R. (2021). What the guide does not tell you: Reflections on and lessons learned from applying the COM-B behavior model for designing real life interventions. Transl. Behav. Med..

[7] Australian Technical Advisory Group on Immunisation (ATAGI) (2024). ATAGI Statement on Administration of COVID-19 Vaccines in 2024. Department of Health, Disability and Ageing. https://www.health.gov.au/resources/publications/atagi-statement-on-the-administration-of-covid-19-vaccines-in-2024?language=en.

[8] Allard N.L., Canevari J., Haslett N., Cowie B. (2023). Access to oral COVID-19 antivirals in the community. Med. J. Aust..

[9] Australian Bureau of Statistics (2023). National Health Survey: Chronic Conditions, 2022–23. https://www.abs.gov.au/statistics/health/health-conditions-and-risks/national-health-survey/latest-release#health-conditions.

[10] Lu P.J., Hung M.C., Jackson H.L., Kriss J.L., Srivastav A., Yankey D., Santibanez T.A., Lee J.T., Meng L., Razzaghi H. (2022). COVID-19 Vaccination and Intent for Vaccination of Adults with Reported Medical Conditions. Am. J. Prev. Med..

[11] Haldane V., De Foo C., Abdalla S.M., Jung A.S., Tan M., Wu S., Chua A., Verma M., Shrestha P., Singh S. (2021). Health systems resilience in managing the COVID-19 pandemic: Lessons from 28 countries. Nat. Med..

[12] Tavakol M., Dennick R. (2011). Making sense of Cronbach’s alpha. Int. J. Med. Educ..

[13] Keyworth C., Epton T., Goldthorpe J., Calam R., Armitage C.J. (2020). Acceptability, reliability, and validity of a brief measure of capabilities, opportunities, and motivations (“COM-B”). Br. J. Health Psychol..

[14] Edwards B., Biddle N., Gray M., Sollis K. (2021). COVID-19 vaccine hesitancy and resistance: Correlates in a nationally representative longitudinal survey of the Australian population. PLoS ONE.

[15] Enticott J., Gill S., Bacon S.L., Lavoie K.L., Epstein D.S., Dawadi S., Teede H.J., Boyle J. (2022). Attitudes towards vaccines and intention to vaccinate against COVID-19: A cross-sectional analysis—Implications for public health communications in Australia. BMJ Open.

[16] Soh S., Ayton D., Bevins A., Skouteris H., Trent M., MacIntyre R. (2024). Attitudes and behaviours regarding COVID-19 mitigation strategies in Australians with an underlying health condition: A cross-sectional study. Health Expect..

[17] Williams C.T., Saini B., Zaidi S.T.R., Kali C., Moujalli G., Castelino R. (2024). Factors influencing COVID-19 vaccine confidence and uptake in Australian adults. Vaccines.

[18] Baker E., Lester L.H., Bentley R., Beer A. (2016). Poor housing quality: Prevalence and health effects. J. Prev. Interv. Community.

[19] Bentley R., Baker E., Mason K., Subramanian S.V., Kavanagh A.M. (2011). Association between housing affordability and mental health: A longitudinal analysis of a nationally representative household survey in Australia. Am. J. Epidemiol..

[20] Doherty I.A., Pilkington W., Brown L., Billings V., Hoffler U., Paulin L., Kimbro K.S., Baker B., Zhang T., Locklear T. (2021). COVID-19 vaccine hesitancy in underserved communities of North Carolina. PLoS ONE.

[21] Petherick A., Goldszmidt R., Andrade E.B., Furst R., Hale T., Pott A., Wood A. (2021). A worldwide assessment of COVID-19 pandemic-policy fatigue. Lancet.

[22] Lazarus J.V., Ratzan S.C., Palayew A., Gostin L.O., Larson H.J., Rabin K., Kimball S., El-Mohandes A. (2021). A global survey of potential acceptance of a COVID-19 vaccine. Nat. Med..

[23] Sherman S.M., Smith L.E., Sim J., Amlôt R., Cutts M., Dasch H., Rubin G.J., Sevdalis N. (2021). COVID-19 vaccination intention in the UK: Results from the COVID-19 vaccination acceptability study (CoVAccS), a nationally representative cross-sectional survey. Hum. Vaccines Immunother..

[24] Nga N.T.V., Xuan V.N., Trong V.A., Thao P.H., Doanh D.C. (2023). Perceived Barriers and Intentions to Receive COVID-19 Vaccines: Psychological Distress as a Moderator. Vaccines.

[25] Betsch C., Schmid P., Heinemeier D., Korn L., Holtmann C., Böhm R. (2018). Beyond confidence: Development of a measure assessing the 5C psychological antecedents of vaccination. PLoS ONE.

[26] Razai M.S., Oakeshott P., Esmail A., Wiysonge C.S., Viswanath K., Mills M.C. (2021). COVID-19 vaccine hesitancy among ethnic minority groups. BMJ.

[27] Kleitman S., Fullerton D.J., Law M.K.H., Blanchard M.D., Campbell R., Tait M.-A., Schulz J., Lee J., Stankov L., King M.T. (2023). The Psychology of COVID-19 Booster Hesitancy, Acceptance and Resistance in Australia. Vaccines.

[28] Oyegun E., Ategbole M., Jorgensen C., Fisher A., Briggs M.H., Gutekunst L., Roberts E., Koumans E.H. (2025). Factors associated with knowledge, attitudes, and behaviors regarding antiviral medications for COVID-19 among US adults. PLoS ONE.

