# Supplementary material for: Behavioural Drivers of COVID-19 Vaccination and Antiviral Uptake in Australia: A Cross-Sectional Analysis Using the COM-B Framework
Source: Vaccines (Basel). 2026 May 31;14(6):495. doi: 10.3390/vaccines14060495 (PMC13308425; doi:10.3390/vaccines14060495)
Supplement: Supplementary file 1 [file vaccines-14-00495-s001.zip › vaccines-4294772-supplementary.pdf]

Supplementary Table S1

Table: LASSO Standardised Coefficients — Selected Predictors by Outcome

Outcome	Predictor	LASSO Coefficient	Direction	Selected
Antiviral uptake (Q33)	Q85 seek antivirals	-0.8526	↓ Negative	Yes
Antiviral uptake (Q33)	Any Q32 risk factor	0.2974	↑ Positive	Yes
Antiviral uptake (Q33)	Q86 take antivirals	0.2604	↑ Positive	Yes
Antiviral uptake (Q33)	Risk stacked	0.2422	↑ Positive	Yes
Antiviral uptake (Q33)	Age	0.2125	↑ Positive	Yes
Antiviral uptake (Q33)	Q79 vax prevents infection	-0.209	↓ Negative	Yes
Antiviral uptake (Q33)	Any chronic condition	-0.1514	↓ Negative	Yes
Antiviral uptake (Q33)	Renting	-0.1354	↓ Negative	Yes
Antiviral uptake (Q33)	Obesity	0.1295	↑ Positive	Yes
Antiviral uptake (Q33)	Heart disease	0.0961	↑ Positive	Yes
Antiviral uptake (Q33)	Currently employed	-0.094	↓ Negative	Yes
Antiviral uptake (Q33)	Asthma/Respiratory	0.0847	↑ Positive	Yes
Antiviral uptake (Q33)	Non-English at home	0.0697	↑ Positive	Yes
Antiviral uptake (Q33)	Q78 vax reduces risk	0.0668	↑ Positive	Yes
Antiviral uptake (Q33)	Healthcare worker	-0.0599	↓ Negative	Yes
Antiviral uptake (Q33)	Female gender	-0.054	↓ Negative	Yes
Antiviral uptake (Q33)	Tertiary education	0.0446	↑ Positive	Yes
Antiviral uptake (Q33)	Cancer	0.0383	↑ Positive	Yes
Antiviral uptake (Q33)	Hypertension	0.0318	↑ Positive	Yes
Antiviral uptake (Q33)	Rural/remote area	-0.0212	↓ Negative	Yes
Antiviral uptake (Q33)	Immunocompromised	0.0204	↑ Positive	Yes
Antiviral uptake (Q33)	Q80 vax reduces severity	0.0173	↑ Positive	Yes
Antiviral uptake (Q33)	Born overseas	0.0162	↑ Positive	Yes

Antiviral uptake (Q33)	Q69 doses received	-0.0064	↓ Negative	Yes
Antiviral uptake (Q33)	Diabetes	0.0039	↑ Positive	Yes
Booster 2023 (Q63)	Q78 vax reduces risk	-0.6795	↓ Negative	Yes
Booster 2023 (Q63)	Q69 doses received	0.4806	↑ Positive	Yes
Booster 2023 (Q63)	Q80 vax reduces severity	-0.4013	↓ Negative	Yes
Booster 2023 (Q63)	Age	0.2734	↑ Positive	Yes
Booster 2023 (Q63)	Q86 take antivirals	0.2322	↑ Positive	Yes
Booster 2023 (Q63)	Q85 seek antivirals	-0.2277	↓ Negative	Yes
Booster 2023 (Q63)	Any Q32 risk factor	0.126	↑ Positive	Yes
Booster 2023 (Q63)	Renting	-0.1161	↓ Negative	Yes
Booster 2023 (Q63)	Q79 vax prevents infection	-0.0999	↓ Negative	Yes
Booster 2023 (Q63)	Risk stacked	0.0774	↑ Positive	Yes
Booster 2023 (Q63)	Healthcare worker	0.067	↑ Positive	Yes
Booster 2023 (Q63)	Female gender	-0.0468	↓ Negative	Yes
Booster 2023 (Q63)	Immunocompromised	0.046	↑ Positive	Yes
Booster 2023 (Q63)	Hypertension	0.0382	↑ Positive	Yes
Booster 2023 (Q63)	Obesity	0.0333	↑ Positive	Yes
Booster 2023 (Q63)	Rural/remote area	-0.0212	↓ Negative	Yes
Booster 2023 (Q63)	Non-English at home	-0.0078	↓ Negative	Yes
Booster 2023 (Q63)	Heart disease	0.0052	↑ Positive	Yes
Booster 2024 (Q66)	Q78 vax reduces risk	-0.6926	↓ Negative	Yes
Booster 2024 (Q66)	Q69 doses received	0.6345	↑ Positive	Yes
Booster 2024 (Q66)	Q85 seek antivirals	-0.3455	↓ Negative	Yes
Booster 2024 (Q66)	Q79 vax prevents infection	-0.3216	↓ Negative	Yes
Booster 2024 (Q66)	Renting	-0.2214	↓ Negative	Yes
Booster 2024 (Q66)	Risk stacked	0.1935	↑ Positive	Yes
Booster 2024 (Q66)	Any Q32 risk factor	0.1793	↑ Positive	Yes
Booster 2024 (Q66)	Q80 vax reduces severity	-0.1354	↓ Negative	Yes
Booster 2024 (Q66)	Healthcare worker	0.1305	↑ Positive	Yes

Booster 2024 (Q66)	Female gender	-0.1252	↓ Negative	Yes
Booster 2024 (Q66)	Any chronic condition	-0.1252	↓ Negative	Yes
Booster 2024 (Q66)	Age	0.1126	↑ Positive	Yes
Booster 2024 (Q66)	Currently employed	-0.0954	↓ Negative	Yes
Booster 2024 (Q66)	Tertiary education	0.0812	↑ Positive	Yes
Booster 2024 (Q66)	Non-English at home	-0.0715	↓ Negative	Yes
Booster 2024 (Q66)	Born overseas	-0.0711	↓ Negative	Yes
Booster 2024 (Q66)	Asthma/Respiratory	0.0697	↑ Positive	Yes
Booster 2024 (Q66)	Heart disease	0.0549	↑ Positive	Yes
Booster 2024 (Q66)	Rural/remote area	-0.0361	↓ Negative	Yes
Booster 2024 (Q66)	Obesity	0.0357	↑ Positive	Yes
Booster 2024 (Q66)	Cancer	0.0345	↑ Positive	Yes
Booster 2024 (Q66)	Q86 take antivirals	0.0332	↑ Positive	Yes
Booster 2024 (Q66)	Diabetes	-0.0183	↓ Negative	Yes
Booster 2024 (Q66)	Hypertension	-0.0008	↓ Negative	Yes
Booster 2024 (Q66)	Immunocompromised	0.0005	↑ Positive	Yes
Intention: new booster (Q74)	Q86 take antivirals	1.1966	↑ Positive	Yes
Intention: new booster (Q74)	Q79 vax prevents infection	-0.6407	↓ Negative	Yes
Intention: new booster (Q74)	Age	0.2226	↑ Positive	Yes
Intention: new booster (Q74)	Q69 doses received	0.1994	↑ Positive	Yes
Intention: new booster (Q74)	Q85 seek antivirals	-0.1191	↓ Negative	Yes
Intention: new booster (Q74)	Renting	-0.1174	↓ Negative	Yes
Intention: new booster (Q74)	Tertiary education	0.1123	↑ Positive	Yes
Intention: new booster (Q74)	Currently employed	-0.0929	↓ Negative	Yes
Intention: new booster (Q74)	Hypertension	0.089	↑ Positive	Yes
Intention: new booster (Q74)	Obesity	0.0685	↑ Positive	Yes
Intention: new booster (Q74)	Non-English at home	-0.044	↓ Negative	Yes
Intention: new booster (Q74)	Healthcare worker	0.0397	↑ Positive	Yes
Intention: new booster (Q74)	Rural/remote area	-0.0329	↓ Negative	Yes

Intention: new booster (Q74)	Cancer	0.0314	↑ Positive	Yes
Intention: new booster (Q74)	Any chronic condition	0.0269	↑ Positive	Yes
Intention: new booster (Q74)	Female gender	-0.0257	↓ Negative	Yes
Intention: new booster (Q74)	Risk stacked	0.0187	↑ Positive	Yes
Intention: new booster (Q74)	Born overseas	0.0109	↑ Positive	Yes
Annual booster intention (Q77)	Q86 take antivirals	1.1419	↑ Positive	Yes
Annual booster intention (Q77)	Q79 vax prevents infection	-0.7251	↓ Negative	Yes
Annual booster intention (Q77)	Age	0.2263	↑ Positive	Yes
Annual booster intention (Q77)	Q69 doses received	0.2212	↑ Positive	Yes
Annual booster intention (Q77)	Q85 seek antivirals	-0.1558	↓ Negative	Yes
Annual booster intention (Q77)	Renting	-0.1081	↓ Negative	Yes
Annual booster intention (Q77)	Currently employed	-0.0929	↓ Negative	Yes
Annual booster intention (Q77)	Healthcare worker	0.0875	↑ Positive	Yes
Annual booster intention (Q77)	Tertiary education	0.079	↑ Positive	Yes
Annual booster intention (Q77)	Risk stacked	0.0743	↑ Positive	Yes
Annual booster intention (Q77)	Obesity	0.0664	↑ Positive	Yes
Annual booster intention (Q77)	Non-English at home	-0.0619	↓ Negative	Yes
Annual booster intention (Q77)	Diabetes	0.0569	↑ Positive	Yes
Annual booster intention (Q77)	Hypertension	0.0485	↑ Positive	Yes
Annual booster intention (Q77)	Rural/remote area	-0.0429	↓ Negative	Yes
Annual booster intention (Q77)	Born overseas	0.0409	↑ Positive	Yes
Annual booster intention (Q77)	Heart disease	0.0201	↑ Positive	Yes
Annual booster intention (Q77)	Asthma/Respiratory	0.0166	↑ Positive	Yes
Willingness: antivirals (Q35)	Q78 vax reduces risk	-0.5735	↓ Negative	Yes
Willingness: antivirals (Q35)	Q85 seek antivirals	-0.3613	↓ Negative	Yes
Willingness: antivirals (Q35)	Q80 vax reduces severity	-0.3052	↓ Negative	Yes
Willingness: antivirals (Q35)	Age	0.2279	↑ Positive	Yes
Willingness: antivirals (Q35)	Any chronic condition	0.1045	↑ Positive	Yes
Willingness: antivirals (Q35)	Q79 vax prevents infection	0.0866	↑ Positive	Yes

Willingness: antivirals (Q35)	Healthcare worker	-0.077	↓ Negative	Yes
Willingness: antivirals (Q35)	Any Q32 risk factor	0.0606	↑ Positive	Yes
Willingness: antivirals (Q35)	Hypertension	0.054	↑ Positive	Yes
Willingness: antivirals (Q35)	Immunocompromised	0.0512	↑ Positive	Yes
Willingness: antivirals (Q35)	Female gender	0.0445	↑ Positive	Yes
Willingness: antivirals (Q35)	Currently employed	-0.0385	↓ Negative	Yes
Willingness: antivirals (Q35)	Rural/remote area	-0.037	↓ Negative	Yes
Willingness: antivirals (Q35)	Heart disease	0.0332	↑ Positive	Yes
Willingness: antivirals (Q35)	Asthma/Respiratory	0.0279	↑ Positive	Yes
Willingness: antivirals (Q35)	Obesity	0.023	↑ Positive	Yes
Willingness: antivirals (Q35)	Tertiary education	0.008	↑ Positive	Yes
Willingness: antivirals (Q35)	Cancer	0.0048	↑ Positive	Yes
Willingness: antivirals (Q35)	Q69 doses received	0.004	↑ Positive	Yes
Seek antivirals from GP (Q85)	Q86 take antivirals	-0.168	↓ Negative	Yes
Seek antivirals from GP (Q85)	Q78 vax reduces risk	-0.1025	↓ Negative	Yes
Seek antivirals from GP (Q85)	Female gender	-0.0945	↓ Negative	Yes
Seek antivirals from GP (Q85)	Diabetes	0.081	↑ Positive	Yes
Seek antivirals from GP (Q85)	Hypertension	0.0775	↑ Positive	Yes
Seek antivirals from GP (Q85)	Immunocompromised	0.0759	↑ Positive	Yes
Seek antivirals from GP (Q85)	Non-English at home	-0.0557	↓ Negative	Yes
Seek antivirals from GP (Q85)	Q69 doses received	0.0525	↑ Positive	Yes
Seek antivirals from GP (Q85)	Tertiary education	0.0514	↑ Positive	Yes
Seek antivirals from GP (Q85)	Obesity	0.0465	↑ Positive	Yes
Seek antivirals from GP (Q85)	Born overseas	-0.0402	↓ Negative	Yes
Seek antivirals from GP (Q85)	Age	0.0401	↑ Positive	Yes
Seek antivirals from GP (Q85)	Currently employed	-0.0356	↓ Negative	Yes
Seek antivirals from GP (Q85)	Healthcare worker	0.0345	↑ Positive	Yes
Seek antivirals from GP (Q85)	Risk stacked	-0.0334	↓ Negative	Yes
Seek antivirals from GP (Q85)	Cancer	0.0306	↑ Positive	Yes

Seek antivirals from GP (Q85)	Any Q32 risk factor	0.0277	↑ Positive	Yes
Seek antivirals from GP (Q85)	Q80 vax reduces severity	0.0255	↑ Positive	Yes
Seek antivirals from GP (Q85)	Renting	-0.0218	↓ Negative	Yes
Seek antivirals from GP (Q85)	Heart disease	0.0204	↑ Positive	Yes
Seek antivirals from GP (Q85)	Q79 vax prevents infection	0.0187	↑ Positive	Yes
Seek antivirals from GP (Q85)	Rural/remote area	-0.0185	↓ Negative	Yes
Seek antivirals from GP (Q85)	Asthma/Respiratory	0.0127	↑ Positive	Yes
Take antivirals if eligible (Q86)	Q78 vax reduces risk	-0.6272	↓ Negative	Yes
Take antivirals if eligible (Q86)	Q85 seek antivirals	-0.4142	↓ Negative	Yes
Take antivirals if eligible (Q86)	Age	0.3816	↑ Positive	Yes
Take antivirals if eligible (Q86)	Q80 vax reduces severity	-0.3809	↓ Negative	Yes
Take antivirals if eligible (Q86)	Any chronic condition	0.2329	↑ Positive	Yes
Take antivirals if eligible (Q86)	Risk stacked	-0.1705	↓ Negative	Yes
Take antivirals if eligible (Q86)	Q79 vax prevents infection	0.1459	↑ Positive	Yes
Take antivirals if eligible (Q86)	Currently employed	-0.0993	↓ Negative	Yes
Take antivirals if eligible (Q86)	Healthcare worker	-0.0969	↓ Negative	Yes
Take antivirals if eligible (Q86)	Any Q32 risk factor	0.0718	↑ Positive	Yes
Take antivirals if eligible (Q86)	Tertiary education	0.0591	↑ Positive	Yes
Take antivirals if eligible (Q86)	Non-English at home	0.0516	↑ Positive	Yes
Take antivirals if eligible (Q86)	Hypertension	0.0468	↑ Positive	Yes
Take antivirals if eligible (Q86)	Cancer	0.0458	↑ Positive	Yes
Take antivirals if eligible (Q86)	Obesity	-0.0442	↓ Negative	Yes
Take antivirals if eligible (Q86)	Female gender	0.0434	↑ Positive	Yes
Take antivirals if eligible (Q86)	Asthma/Respiratory	0.0354	↑ Positive	Yes
Take antivirals if eligible (Q86)	Heart disease	-0.0296	↓ Negative	Yes
Take antivirals if eligible (Q86)	Renting	0.026	↑ Positive	Yes
Take antivirals if eligible (Q86)	Immunocompromised	0.0257	↑ Positive	Yes
Take antivirals if eligible (Q86)	Born overseas	-0.0173	↓ Negative	Yes
Take antivirals if eligible (Q86)	Q69 doses received	0.0035	↑ Positive	Yes

Take antivirals if eligible (Q86)	Rural/remote area	-0.0024	↓ Negative	Yes
Belief: vax reduces risk (Q78)	Q86 take antivirals	0.9063	↑ Positive	Yes
Belief: vax reduces risk (Q78)	Q85 seek antivirals	-0.3454	↓ Negative	Yes
Belief: vax reduces risk (Q78)	Tertiary education	0.1933	↑ Positive	Yes
Belief: vax reduces risk (Q78)	Q69 doses received	-0.1917	↓ Negative	Yes
Belief: vax reduces risk (Q78)	Risk stacked	0.1148	↑ Positive	Yes
Belief: vax reduces risk (Q78)	Diabetes	0.1091	↑ Positive	Yes
Belief: vax reduces risk (Q78)	Female gender	-0.0992	↓ Negative	Yes
Belief: vax reduces risk (Q78)	Any chronic condition	-0.0973	↓ Negative	Yes
Belief: vax reduces risk (Q78)	Born overseas	0.0819	↑ Positive	Yes
Belief: vax reduces risk (Q78)	Renting	-0.0751	↓ Negative	Yes
Belief: vax reduces risk (Q78)	Obesity	-0.0725	↓ Negative	Yes
Belief: vax reduces risk (Q78)	Immunocompromised	-0.0613	↓ Negative	Yes
Belief: vax reduces risk (Q78)	Healthcare worker	0.0456	↑ Positive	Yes
Belief: vax reduces risk (Q78)	Asthma/Respiratory	-0.038	↓ Negative	Yes
Belief: vax reduces risk (Q78)	Cancer	0.0225	↑ Positive	Yes
Belief: vax reduces risk (Q78)	Heart disease	0.0182	↑ Positive	Yes
Belief: vax reduces risk (Q78)	Currently employed	0.0177	↑ Positive	Yes
Belief: vax reduces risk (Q78)	Rural/remote area	-0.0106	↓ Negative	Yes
Belief: vax prevents infection (Q79)	Q85 seek antivirals	-0.4054	↓ Negative	Yes
Belief: vax prevents infection (Q79)	Age	-0.317	↓ Negative	Yes
Belief: vax prevents infection (Q79)	Female gender	-0.2619	↓ Negative	Yes
Belief: vax prevents infection (Q79)	Any Q32 risk factor	0.171	↑ Positive	Yes
Belief: vax prevents infection (Q79)	Non-English at home	0.0967	↑ Positive	Yes
Belief: vax prevents infection (Q79)	Q86 take antivirals	0.0805	↑ Positive	Yes
Belief: vax prevents infection (Q79)	Currently employed	0.0737	↑ Positive	Yes
Belief: vax prevents infection (Q79)	Q69 doses received	-0.0652	↓ Negative	Yes
Belief: vax prevents infection (Q79)	Tertiary education	0.0614	↑ Positive	Yes
Belief: vax prevents infection (Q79)	Any chronic condition	-0.0547	↓ Negative	Yes

Belief: vax prevents infection (Q79)	Hypertension	-0.0528	↓ Negative	Yes
Belief: vax prevents infection (Q79)	Healthcare worker	0.0498	↑ Positive	Yes
Belief: vax prevents infection (Q79)	Diabetes	0.0373	↑ Positive	Yes
Belief: vax prevents infection (Q79)	Born overseas	0.0282	↑ Positive	Yes
Belief: vax prevents infection (Q79)	Immunocompromised	-0.0225	↓ Negative	Yes
Belief: vax prevents infection (Q79)	Renting	-0.0082	↓ Negative	Yes
Belief: vax prevents infection (Q79)	Obesity	0.0032	↑ Positive	Yes
Belief: vax prevents infection (Q79)	Cancer	0.0029	↑ Positive	Yes
Belief: vax reduces severity (Q80)	Q86 take antivirals	0.9273	↑ Positive	Yes
Belief: vax reduces severity (Q80)	Q69 doses received	-0.2729	↓ Negative	Yes
Belief: vax reduces severity (Q80)	Q85 seek antivirals	-0.2004	↓ Negative	Yes
Belief: vax reduces severity (Q80)	Tertiary education	0.1751	↑ Positive	Yes
Belief: vax reduces severity (Q80)	Healthcare worker	0.0997	↑ Positive	Yes
Belief: vax reduces severity (Q80)	Renting	-0.088	↓ Negative	Yes
Belief: vax reduces severity (Q80)	Rural/remote area	-0.0767	↓ Negative	Yes
Belief: vax reduces severity (Q80)	Diabetes	0.0728	↑ Positive	Yes
Belief: vax reduces severity (Q80)	Female gender	-0.0718	↓ Negative	Yes
Belief: vax reduces severity (Q80)	Born overseas	0.0595	↑ Positive	Yes
Belief: vax reduces severity (Q80)	Asthma/Respiratory	-0.0558	↓ Negative	Yes
Belief: vax reduces severity (Q80)	Currently employed	-0.0436	↓ Negative	Yes
Belief: vax reduces severity (Q80)	Risk stacked	0.0308	↑ Positive	Yes
Belief: vax reduces severity (Q80)	Hypertension	0.0287	↑ Positive	Yes
Belief: vax reduces severity (Q80)	Heart disease	-0.0104	↓ Negative	Yes
Belief: vax reduces severity (Q80)	Obesity	0.0043	↑ Positive	Yes
Belief: vax reduces severity (Q80)	Any Q32 risk factor	-0.0035	↓ Negative	Yes
Belief: vax reduces severity (Q80)	Non-English at home	0.0013	↑ Positive	Yes
Boosters important (Q89)	Q86 take antivirals	1.2077	↑ Positive	Yes
Boosters important (Q89)	Q79 vax prevents infection	-0.9134	↓ Negative	Yes
Boosters important (Q89)	Age	0.3897	↑ Positive	Yes

Boosters important (Q89)	Tertiary education	0.1978	↑ Positive	Yes
Boosters important (Q89)	Q69 doses received	0.1551	↑ Positive	Yes
Boosters important (Q89)	Healthcare worker	0.1397	↑ Positive	Yes
Boosters important (Q89)	Q85 seek antivirals	-0.1374	↓ Negative	Yes
Boosters important (Q89)	Risk stacked	0.1319	↑ Positive	Yes
Boosters important (Q89)	Currently employed	-0.0971	↓ Negative	Yes
Boosters important (Q89)	Born overseas	0.082	↑ Positive	Yes
Boosters important (Q89)	Any chronic condition	-0.0678	↓ Negative	Yes
Boosters important (Q89)	Obesity	0.0658	↑ Positive	Yes
Boosters important (Q89)	Non-English at home	0.0628	↑ Positive	Yes
Boosters important (Q89)	Rural/remote area	-0.0521	↓ Negative	Yes
Boosters important (Q89)	Immunocompromised	-0.0493	↓ Negative	Yes
Boosters important (Q89)	Renting	-0.0421	↓ Negative	Yes
Boosters important (Q89)	Female gender	0.0278	↑ Positive	Yes
Boosters important (Q89)	Hypertension	0.0252	↑ Positive	Yes
Boosters important (Q89)	Any Q32 risk factor	-0.0155	↓ Negative	Yes
Boosters important (Q89)	Asthma/Respiratory	-0.0062	↓ Negative	Yes

Supplementary Table S2

Univariate Logistic Regression Results

Outcome	Predictor	N	n (outcome)	OR	CI Lower	CI Upper	95% CI	p-value
Antiviral uptake Q33	Age (continuous)	2576	391	1.032	1.026	1.039	1.03 (1.03–1.04)	<0.001
	Female gender	2564	390	0.491	0.394	0.611	0.49 (0.39–0.61)	<0.001
	Born overseas	2576	391	1.033	0.794	1.345	1.03 (0.79–1.34)	0.808
	Non-English at home	2575	390	0.913	0.639	1.305	0.91 (0.64–1.31)	0.619
	Tertiary education	2576	391	1.146	0.923	1.422	1.15 (0.92–1.42)	0.216
	Currently employed	2575	390	0.505	0.405	0.628	0.50 (0.41–0.63)	<0.001
	Healthcare/first responder	2575	390	1.022	0.777	1.343	1.02 (0.78–1.34)	0.879
	Renting	2575	390	0.496	0.39	0.632	0.50 (0.39–0.63)	<0.001
	Rural/remote area	2574	390	0.994	0.651	1.52	0.99 (0.65–1.52)	0.979
	Any chronic condition	2576	391	2.311	1.763	3.029	2.31 (1.76–3.03)	<0.001
	Diabetes	2576	391	3.094	2.328	4.113	3.09 (2.33–4.11)	<0.001
	Hypertension	2576	391	2.667	2.113	3.365	2.67 (2.11–3.37)	<0.001
	Heart disease	2576	391	4.18	3.008	5.809	4.18 (3.01–5.81)	<0.001
	Cancer	2576	391	2.862	2.073	3.95	2.86 (2.07–3.95)	<0.001
	Asthma/Respiratory	2576	391	1.806	1.431	2.279	1.81 (1.43–2.28)	<0.001
	Immunocompromised	2576	391	2.599	1.553	4.35	2.60 (1.55–4.35)	<0.001
	Obesity	2576	391	2.846	1.815	4.463	2.85 (1.81–4.46)	<0.001
	Any Q32 risk factor	2576	391	4.143	3.316	5.177	4.14 (3.32–5.18)	<0.001
	Risk stacked	2576	391	4.055	2.867	5.737	4.06 (2.87–5.74)	<0.001
	Motivation score (per SD)	2576	391	1.973	1.729	2.252	1.97 (1.73–2.25)	<0.001

	Opportunity score (per SD)	2576	391	1.297	1.162	1.448	1.30 (1.16–1.45)	<0.001
Booster 2023 Q63	Age (continuous)	5027	2552	1.036	1.033	1.04	1.04 (1.03–1.04)	<0.001
	Female gender	5001	2546	0.565	0.504	0.634	0.57 (0.50–0.63)	<0.001
	Born overseas	5027	2552	1.155	1.013	1.317	1.15 (1.01–1.32)	0.032
	Non-English at home	5026	2552	0.797	0.67	0.948	0.80 (0.67–0.95)	0.011
	Tertiary education	5026	2551	1.101	0.984	1.232	1.10 (0.98–1.23)	0.094
	Currently employed	5026	2552	0.524	0.467	0.588	0.52 (0.47–0.59)	<0.001
	Healthcare/first responder	5026	2552	1.104	0.945	1.29	1.10 (0.94–1.29)	0.213
	Renting	5026	2552	0.517	0.46	0.58	0.52 (0.46–0.58)	<0.001
	Rural/remote area	5023	2551	0.931	0.76	1.14	0.93 (0.76–1.14)	0.488
	Any chronic condition	5027	2552	1.946	1.729	2.189	1.95 (1.73–2.19)	<0.001
	Diabetes	5027	2552	2.26	1.878	2.718	2.26 (1.88–2.72)	<0.001
	Hypertension	5027	2552	2.524	2.193	2.906	2.52 (2.19–2.91)	<0.001
	Heart disease	5027	2552	2.459	1.946	3.108	2.46 (1.95–3.11)	<0.001
	Cancer	5027	2552	2.487	2.016	3.068	2.49 (2.02–3.07)	<0.001
	Asthma/Respiratory	5027	2552	1.203	1.049	1.381	1.20 (1.05–1.38)	0.008
	Immunocompromised	5027	2552	2.298	1.557	3.392	2.30 (1.56–3.39)	<0.001
	Obesity	5027	2552	1.462	1.047	2.043	1.46 (1.05–2.04)	0.026
	Any Q32 risk factor	5027	2552	1.978	1.738	2.251	1.98 (1.74–2.25)	<0.001
	Risk stacked	5027	2552	2.278	2.011	2.58	2.28 (2.01–2.58)	<0.001
	Motivation score (per SD)	5027	2552	1.88	1.765	2.002	1.88 (1.76–2.00)	<0.001
	Opportunity score (per SD)	5027	2552	1.107	1.048	1.171	1.11 (1.05–1.17)	<0.001
Booster 2024 Q66	Age (continuous)	5098	974	1.035	1.031	1.04	1.04 (1.03–1.04)	<0.001
	Female gender	5071	970	0.481	0.417	0.554	0.48 (0.42–0.55)	<0.001
	Born overseas	5098	974	0.909	0.768	1.076	0.91 (0.77–1.08)	0.267

	Non-English at home	5097	974	0.596	0.463	0.766	0.60 (0.46–0.77)	<0.001
	Tertiary education	5097	974	1.136	0.987	1.308	1.14 (0.99–1.31)	0.075
	Currently employed	5097	974	0.473	0.411	0.545	0.47 (0.41–0.54)	<0.001
	Healthcare/first responder	5097	974	1.232	1.021	1.487	1.23 (1.02–1.49)	0.030
	Renting	5097	974	0.44	0.375	0.516	0.44 (0.38–0.52)	<0.001
	Rural/remote area	5094	973	1.043	0.811	1.343	1.04 (0.81–1.34)	0.741
	Any chronic condition	5098	974	1.985	1.689	2.333	1.98 (1.69–2.33)	<0.001
	Diabetes	5098	974	2.172	1.794	2.63	2.17 (1.79–2.63)	<0.001
	Hypertension	5098	974	2.186	1.875	2.549	2.19 (1.87–2.55)	<0.001
	Heart disease	5098	974	2.777	2.214	3.484	2.78 (2.21–3.48)	<0.001
	Cancer	5098	974	2.314	1.877	2.852	2.31 (1.88–2.85)	<0.001
	Asthma/Respiratory	5098	974	1.459	1.238	1.718	1.46 (1.24–1.72)	<0.001
	Immunocompromised	5098	974	2.035	1.388	2.983	2.04 (1.39–2.98)	<0.001
	Obesity	5098	974	1.551	1.068	2.252	1.55 (1.07–2.25)	0.021
	Any Q32 risk factor	5098	974	2.383	2.057	2.761	2.38 (2.06–2.76)	<0.001
	Risk stacked	5098	974	2.745	2.279	3.307	2.75 (2.28–3.31)	<0.001
	Motivation score (per SD)	5098	974	2.867	2.618	3.139	2.87 (2.62–3.14)	<0.001
	Opportunity score (per SD)	5098	974	1.017	0.948	1.09	1.02 (0.95–1.09)	0.643
Intention new booster Q74	Age (continuous)	4158	2515	1.033	1.029	1.036	1.03 (1.03–1.04)	<0.001
	Female gender	4134	2506	0.58	0.509	0.661	0.58 (0.51–0.66)	<0.001
	Born overseas	4158	2515	1.181	1.017	1.372	1.18 (1.02–1.37)	0.029
	Non-English at home	4158	2515	0.795	0.655	0.966	0.80 (0.66–0.97)	0.021
	Tertiary education	4157	2514	1.317	1.16	1.495	1.32 (1.16–1.50)	<0.001
	Currently employed	4158	2515	0.513	0.45	0.585	0.51 (0.45–0.59)	<0.001

	Healthcare/first responder	4158	2515	0.999	0.841	1.186	1.00 (0.84–1.19)	0.991
	Renting	4158	2515	0.531	0.467	0.603	0.53 (0.47–0.60)	<0.001
	Rural/remote area	4156	2514	0.849	0.679	1.061	0.85 (0.68–1.06)	0.150
	Any chronic condition	4158	2515	1.889	1.657	2.153	1.89 (1.66–2.15)	<0.001
	Diabetes	4158	2515	2.178	1.757	2.699	2.18 (1.76–2.70)	<0.001
	Hypertension	4158	2515	2.576	2.185	3.036	2.58 (2.19–3.04)	<0.001
	Heart disease	4158	2515	2.156	1.657	2.804	2.16 (1.66–2.80)	<0.001
	Cancer	4158	2515	2.838	2.193	3.674	2.84 (2.19–3.67)	<0.001
	Asthma/Respiratory	4158	2515	1.193	1.022	1.393	1.19 (1.02–1.39)	0.026
	Immunocompromised	4158	2515	1.871	1.21	2.892	1.87 (1.21–2.89)	0.005
	Obesity	4158	2515	1.586	1.08	2.327	1.59 (1.08–2.33)	0.019
	Any Q32 risk factor	4158	2515	1.758	1.521	2.033	1.76 (1.52–2.03)	<0.001
	Risk stacked	4158	2515	2.058	1.794	2.36	2.06 (1.79–2.36)	<0.001
	Motivation score (per SD)	4158	2515	1.887	1.757	2.027	1.89 (1.76–2.03)	<0.001
	Opportunity score (per SD)	4158	2515	1.249	1.173	1.329	1.25 (1.17–1.33)	<0.001
Annual booster intention Q77	Age (continuous)	4227	2544	1.032	1.028	1.035	1.03 (1.03–1.04)	<0.001
	Female gender	4207	2535	0.595	0.523	0.677	0.59 (0.52–0.68)	<0.001
	Born overseas	4227	2544	1.208	1.041	1.402	1.21 (1.04–1.40)	0.013
	Non-English at home	4227	2544	0.802	0.66	0.975	0.80 (0.66–0.97)	0.027
	Tertiary education	4226	2544	1.256	1.107	1.424	1.26 (1.11–1.42)	<0.001
	Currently employed	4227	2544	0.516	0.454	0.588	0.52 (0.45–0.59)	<0.001
	Healthcare/first responder	4227	2544	1.113	0.936	1.324	1.11 (0.94–1.32)	0.226
	Renting	4227	2544	0.544	0.48	0.618	0.54 (0.48–0.62)	<0.001
	Rural/remote area	4225	2543	0.911	0.73	1.137	0.91 (0.73–1.14)	0.408

	Any chronic condition	4227	2544	1.879	1.651	2.14	1.88 (1.65–2.14)	<0.001
	Diabetes	4227	2544	2.453	1.974	3.048	2.45 (1.97–3.05)	<0.001
	Hypertension	4227	2544	2.458	2.09	2.891	2.46 (2.09–2.89)	<0.001
	Heart disease	4227	2544	2.242	1.725	2.914	2.24 (1.73–2.91)	<0.001
	Cancer	4227	2544	2.546	1.99	3.257	2.55 (1.99–3.26)	<0.001
	Asthma/Respiratory	4227	2544	1.249	1.07	1.459	1.25 (1.07–1.46)	0.005
	Immunocompromised	4227	2544	1.789	1.17	2.734	1.79 (1.17–2.73)	0.007
	Obesity	4227	2544	1.546	1.056	2.263	1.55 (1.06–2.26)	0.025
	Any Q32 risk factor	4227	2544	1.862	1.61	2.153	1.86 (1.61–2.15)	<0.001
	Risk stacked	4227	2544	2.109	1.842	2.415	2.11 (1.84–2.42)	<0.001
	Motivation score (per SD)	4227	2544	1.932	1.798	2.075	1.93 (1.80–2.07)	<0.001
	Opportunity score (per SD)	4227	2544	1.244	1.168	1.324	1.24 (1.17–1.32)	<0.001
Willingness antivirals Q35	Age (continuous)	5177	3357	1.024	1.021	1.027	1.02 (1.02–1.03)	<0.001
	Female gender	5146	3341	0.786	0.698	0.886	0.79 (0.70–0.89)	<0.001
	Born overseas	5177	3357	1.127	0.983	1.292	1.13 (0.98–1.29)	0.087
	Non-English at home	5176	3357	0.868	0.728	1.034	0.87 (0.73–1.03)	0.113
	Tertiary education	5176	3357	1.133	1.009	1.273	1.13 (1.01–1.27)	0.035
	Currently employed	5176	3357	0.604	0.536	0.682	0.60 (0.54–0.68)	<0.001
	Healthcare/first responder	5176	3357	0.828	0.708	0.97	0.83 (0.71–0.97)	0.019
	Renting	5176	3357	0.707	0.629	0.794	0.71 (0.63–0.79)	<0.001
	Rural/remote area	5173	3355	0.836	0.682	1.025	0.84 (0.68–1.02)	0.085
	Any chronic condition	5177	3357	1.879	1.669	2.115	1.88 (1.67–2.12)	<0.001
	Diabetes	5177	3357	2.054	1.675	2.518	2.05 (1.68–2.52)	<0.001
	Hypertension	5177	3357	2.2	1.887	2.565	2.20 (1.89–2.57)	<0.001
	Heart disease	5177	3357	2.186	1.687	2.833	2.19 (1.69–2.83)	<0.001
	Cancer	5177	3357	2.13	1.691	2.684	2.13 (1.69–2.68)	<0.001

	Asthma/Respiratory	5177	3357	1.309	1.132	1.515	1.31 (1.13–1.52)	<0.001
	Immunocompromised	5177	3357	2.345	1.498	3.672	2.35 (1.50–3.67)	<0.001
	Obesity	5177	3357	1.508	1.045	2.175	1.51 (1.04–2.17)	0.028
	Any Q32 risk factor	5177	3357	1.746	1.522	2.003	1.75 (1.52–2.00)	<0.001
	Risk stacked	5177	3357	1.928	1.705	2.179	1.93 (1.71–2.18)	<0.001
	Motivation score (per SD)	5177	3357	2.152	2.016	2.299	2.15 (2.02–2.30)	<0.001
	Opportunity score (per SD)	5177	3357	0.958	0.905	1.014	0.96 (0.90–1.01)	0.138
Seek antivirals from GP Q85	Age (continuous)	5177	3538	1.006	1.003	1.009	1.01 (1.00–1.01)	<0.001
	Female gender	5146	3518	0.774	0.685	0.876	0.77 (0.69–0.88)	<0.001
	Born overseas	5177	3538	0.883	0.77	1.013	0.88 (0.77–1.01)	0.076
	Non-English at home	5176	3538	0.787	0.659	0.939	0.79 (0.66–0.94)	0.008
	Tertiary education	5176	3537	1.058	0.939	1.192	1.06 (0.94–1.19)	0.351
	Currently employed	5176	3538	0.886	0.785	0.999	0.89 (0.79–1.00)	0.049
	Healthcare/first responder	5176	3538	1.076	0.911	1.27	1.08 (0.91–1.27)	0.387
	Renting	5176	3538	0.885	0.785	0.997	0.88 (0.78–1.00)	0.045
	Rural/remote area	5173	3535	0.975	0.789	1.206	0.98 (0.79–1.21)	0.819
	Any chronic condition	5177	3538	1.128	0.998	1.275	1.13 (1.00–1.27)	0.053
	Diabetes	5177	3538	1.505	1.234	1.835	1.51 (1.23–1.84)	<0.001
	Hypertension	5177	3538	1.344	1.16	1.556	1.34 (1.16–1.56)	<0.001
	Heart disease	5177	3538	1.359	1.068	1.73	1.36 (1.07–1.73)	0.013
	Cancer	5177	3538	1.271	1.026	1.575	1.27 (1.03–1.57)	0.028
	Asthma/Respiratory	5177	3538	1.03	0.89	1.192	1.03 (0.89–1.19)	0.690
	Immunocompromised	5177	3538	1.804	1.167	2.789	1.80 (1.17–2.79)	0.008
	Obesity	5177	3538	1.428	0.981	2.08	1.43 (0.98–2.08)	0.063
	Any Q32 risk factor	5177	3538	1.221	1.066	1.399	1.22 (1.07–1.40)	0.004

	Risk stacked	5177	3538	1.12	0.986	1.272	1.12 (0.99–1.27)	0.080
	Motivation score (per SD)	5177	3538	1.008	0.95	1.069	1.01 (0.95–1.07)	0.795
	Opportunity score (per SD)	5177	3538	0.949	0.895	1.006	0.95 (0.90–1.01)	0.081
Take antivirals if eligible Q8	Age (continuous)	5177	3406	1.029	1.026	1.033	1.03 (1.03–1.03)	<0.001
	Female gender	5146	3388	0.732	0.649	0.826	0.73 (0.65–0.83)	<0.001
	Born overseas	5177	3406	1.196	1.041	1.374	1.20 (1.04–1.37)	0.011
	Non-English at home	5176	3406	0.991	0.828	1.185	0.99 (0.83–1.18)	0.917
	Tertiary education	5176	3405	1.182	1.051	1.329	1.18 (1.05–1.33)	0.005
	Currently employed	5176	3406	0.535	0.474	0.605	0.54 (0.47–0.60)	<0.001
	Healthcare/first responder	5176	3406	0.778	0.665	0.911	0.78 (0.66–0.91)	0.002
	Renting	5176	3406	0.702	0.624	0.789	0.70 (0.62–0.79)	<0.001
	Rural/remote area	5173	3404	0.931	0.757	1.146	0.93 (0.76–1.15)	0.500
	Any chronic condition	5177	3406	1.817	1.614	2.047	1.82 (1.61–2.05)	<0.001
	Diabetes	5177	3406	2.026	1.65	2.489	2.03 (1.65–2.49)	<0.001
	Hypertension	5177	3406	2.296	1.963	2.685	2.30 (1.96–2.68)	<0.001
	Heart disease	5177	3406	1.827	1.422	2.347	1.83 (1.42–2.35)	<0.001
	Cancer	5177	3406	2.5	1.959	3.19	2.50 (1.96–3.19)	<0.001
	Asthma/Respiratory	5177	3406	1.243	1.074	1.439	1.24 (1.07–1.44)	0.003
	Immunocompromised	5177	3406	1.844	1.207	2.818	1.84 (1.21–2.82)	0.005
	Obesity	5177	3406	0.979	0.696	1.378	0.98 (0.70–1.38)	0.905
	Any Q32 risk factor	5177	3406	1.574	1.373	1.805	1.57 (1.37–1.80)	<0.001
	Risk stacked	5177	3406	1.819	1.608	2.057	1.82 (1.61–2.06)	<0.001
	Motivation score (per SD)	5177	3406	1.946	1.822	2.077	1.95 (1.82–2.08)	<0.001

	Opportunity score (per SD)	5177	3406	0.873	0.824	0.925	0.87 (0.82–0.92)	<0.001
Belief vax reduces risk Q78	Age (continuous)	5177	3914	1.014	1.01	1.018	1.01 (1.01–1.02)	<0.001
	Female gender	5146	3896	0.648	0.565	0.742	0.65 (0.57–0.74)	<0.001
	Born overseas	5177	3914	1.389	1.185	1.627	1.39 (1.18–1.63)	<0.001
	Non-English at home	5176	3914	1.119	0.914	1.37	1.12 (0.91–1.37)	0.277
	Tertiary education	5176	3914	1.67	1.461	1.909	1.67 (1.46–1.91)	<0.001
	Currently employed	5176	3914	0.849	0.745	0.968	0.85 (0.74–0.97)	0.015
	Healthcare/first responder	5176	3914	1.147	0.956	1.377	1.15 (0.96–1.38)	0.141
	Renting	5176	3914	0.651	0.573	0.74	0.65 (0.57–0.74)	<0.001
	Rural/remote area	5173	3912	0.85	0.679	1.062	0.85 (0.68–1.06)	0.153
	Any chronic condition	5177	3914	1.323	1.161	1.508	1.32 (1.16–1.51)	<0.001
	Diabetes	5177	3914	2.014	1.587	2.555	2.01 (1.59–2.56)	<0.001
	Hypertension	5177	3914	1.581	1.339	1.867	1.58 (1.34–1.87)	<0.001
	Heart disease	5177	3914	1.547	1.174	2.038	1.55 (1.17–2.04)	0.002
	Cancer	5177	3914	1.763	1.363	2.28	1.76 (1.36–2.28)	<0.001
	Asthma/Respiratory	5177	3914	1.019	0.87	1.193	1.02 (0.87–1.19)	0.816
	Immunocompromised	5177	3914	1.081	0.711	1.645	1.08 (0.71–1.65)	0.715
	Obesity	5177	3914	0.797	0.557	1.143	0.80 (0.56–1.14)	0.217
	Any Q32 risk factor	5177	3914	1.445	1.241	1.683	1.45 (1.24–1.68)	<0.001
	Risk stacked	5177	3914	1.449	1.267	1.658	1.45 (1.27–1.66)	<0.001
	Motivation score (per SD)	5177	3914	1.745	1.626	1.873	1.74 (1.63–1.87)	<0.001
	Opportunity score (per SD)	5177	3914	1.134	1.064	1.209	1.13 (1.06–1.21)	<0.001
Belief vax prevents infection	Age (continuous)	5177	1432	0.987	0.983	0.99	0.99 (0.98–0.99)	<0.001

	Female gender	5146	1423	0.594	0.524	0.672	0.59 (0.52–0.67)	<0.001
	Born overseas	5177	1432	1.173	1.018	1.352	1.17 (1.02–1.35)	0.028
	Non-English at home	5176	1432	1.571	1.314	1.879	1.57 (1.31–1.88)	<0.001
	Tertiary education	5176	1432	1.449	1.282	1.638	1.45 (1.28–1.64)	<0.001
	Currently employed	5176	1432	1.45	1.276	1.648	1.45 (1.28–1.65)	<0.001
	Healthcare/first responder	5176	1432	1.43	1.215	1.683	1.43 (1.21–1.68)	<0.001
	Renting	5176	1432	0.957	0.844	1.085	0.96 (0.84–1.08)	0.492
	Rural/remote area	5173	1431	1.028	0.824	1.281	1.03 (0.82–1.28)	0.807
	Any chronic condition	5177	1432	0.863	0.76	0.979	0.86 (0.76–0.98)	0.023
	Diabetes	5177	1432	1.505	1.255	1.804	1.50 (1.26–1.80)	<0.001
	Hypertension	5177	1432	0.805	0.691	0.937	0.80 (0.69–0.94)	0.005
	Heart disease	5177	1432	1.109	0.879	1.399	1.11 (0.88–1.40)	0.384
	Cancer	5177	1432	0.959	0.773	1.189	0.96 (0.77–1.19)	0.701
	Asthma/Respiratory	5177	1432	1.103	0.95	1.281	1.10 (0.95–1.28)	0.197
	Immunocompromised	5177	1432	0.966	0.649	1.438	0.97 (0.65–1.44)	0.864
	Obesity	5177	1432	1.49	1.061	2.091	1.49 (1.06–2.09)	0.021
	Any Q32 risk factor	5177	1432	1.664	1.457	1.901	1.66 (1.46–1.90)	<0.001
	Risk stacked	5177	1432	0.962	0.843	1.099	0.96 (0.84–1.10)	0.573
	Motivation score (per SD)	5177	1432	2.643	2.451	2.85	2.64 (2.45–2.85)	<0.001
	Opportunity score (per SD)	5177	1432	0.953	0.896	1.013	0.95 (0.90–1.01)	0.120
Belief vax reduces severity Q	Age (continuous)	5177	4114	1.013	1.01	1.017	1.01 (1.01–1.02)	<0.001
	Female gender	5146	4094	0.712	0.617	0.823	0.71 (0.62–0.82)	<0.001
	Born overseas	5177	4114	1.34	1.132	1.586	1.34 (1.13–1.59)	<0.001
	Non-English at home	5176	4114	1.161	0.934	1.444	1.16 (0.93–1.44)	0.179
	Tertiary education	5176	4114	1.645	1.426	1.898	1.65 (1.43–1.90)	<0.001

	Currently employed	5176	4114	0.797	0.693	0.917	0.80 (0.69–0.92)	0.002
	Healthcare/first responder	5176	4114	1.298	1.062	1.587	1.30 (1.06–1.59)	0.011
	Renting	5176	4114	0.65	0.567	0.745	0.65 (0.57–0.74)	<0.001
	Rural/remote area	5173	4112	0.691	0.551	0.867	0.69 (0.55–0.87)	0.001
	Any chronic condition	5177	4114	1.396	1.215	1.603	1.40 (1.22–1.60)	<0.001
	Diabetes	5177	4114	1.877	1.458	2.415	1.88 (1.46–2.42)	<0.001
	Hypertension	5177	4114	1.704	1.42	2.043	1.70 (1.42–2.04)	<0.001
	Heart disease	5177	4114	1.271	0.961	1.682	1.27 (0.96–1.68)	0.093
	Cancer	5177	4114	1.574	1.204	2.059	1.57 (1.20–2.06)	<0.001
	Asthma/Respiratory	5177	4114	1.002	0.847	1.184	1.00 (0.85–1.18)	0.985
	Immunocompromised	5177	4114	1.379	0.851	2.235	1.38 (0.85–2.23)	0.192
	Obesity	5177	4114	1.24	0.808	1.902	1.24 (0.81–1.90)	0.326
	Any Q32 risk factor	5177	4114	1.335	1.137	1.567	1.34 (1.14–1.57)	<0.001
	Risk stacked	5177	4114	1.457	1.263	1.679	1.46 (1.26–1.68)	<0.001
	Motivation score (per SD)	5177	4114	1.532	1.425	1.647	1.53 (1.42–1.65)	<0.001
	Opportunity score (per SD)	5177	4114	1.159	1.082	1.241	1.16 (1.08–1.24)	<0.001
Boosters important Q89	Age (continuous)	4216	2815	1.032	1.028	1.036	1.03 (1.03–1.04)	<0.001
	Female gender	4197	2803	0.606	0.53	0.693	0.61 (0.53–0.69)	<0.001
	Born overseas	4216	2815	1.569	1.334	1.846	1.57 (1.33–1.85)	<0.001
	Non-English at home	4216	2815	1.272	1.026	1.576	1.27 (1.03–1.58)	0.028
	Tertiary education	4215	2814	1.513	1.325	1.727	1.51 (1.33–1.73)	<0.001
	Currently employed	4216	2815	0.539	0.471	0.618	0.54 (0.47–0.62)	<0.001
	Healthcare/first responder	4216	2815	1.216	1.01	1.465	1.22 (1.01–1.47)	0.039
	Renting	4216	2815	0.563	0.494	0.642	0.56 (0.49–0.64)	<0.001
	Rural/remote area	4213	2813	0.813	0.647	1.02	0.81 (0.65–1.02)	0.074

	Any chronic condition	4216	2815	1.691	1.479	1.932	1.69 (1.48–1.93)	<0.001
	Diabetes	4216	2815	2.103	1.675	2.641	2.10 (1.67–2.64)	<0.001
	Hypertension	4216	2815	2.329	1.962	2.764	2.33 (1.96–2.76)	<0.001
	Heart disease	4216	2815	2.135	1.611	2.828	2.13 (1.61–2.83)	<0.001
	Cancer	4216	2815	2.426	1.864	3.158	2.43 (1.86–3.16)	<0.001
	Asthma/Respiratory	4216	2815	1.116	0.95	1.31	1.12 (0.95–1.31)	0.183
	Immunocompromised	4216	2815	1.434	0.928	2.216	1.43 (0.93–2.22)	0.104
	Obesity	4216	2815	1.448	0.969	2.163	1.45 (0.97–2.16)	0.071
	Any Q32 risk factor	4216	2815	1.677	1.442	1.951	1.68 (1.44–1.95)	<0.001
	Risk stacked	4216	2815	1.956	1.703	2.246	1.96 (1.70–2.25)	<0.001
	Motivation score (per SD)	4216	2815	2.306	2.13	2.497	2.31 (2.13–2.50)	<0.001
	Opportunity score (per SD)	4216	2815	1.252	1.173	1.336	1.25 (1.17–1.34)	<0.001

Supplementary Table S3

Table: LASSO Penalised Logistic Regression — 10-fold Cross-Validation										
Outcome	N	n (outcome)	Optimal λ	Best C	CV AUC (mean)	CV AUC (SD)	Predictors total	Predictors selected	COM-B items excluded	Selected predictors
Antiviral uptake (Q33)	2562	389	2.811769	0.355648	0.8292	0.0316	25	25		Q85 seek antivirals, Any Q32 risk factor, Q86 take antivirals, Risk stacked, Age, Q79 vax prevents infection, Any chronic condition, Renting, Obesity, Heart disease, Currently employed, Asthma/Respiratory, Non-English at home, Q78 vax reduces risk, Healthcare worker, Female gender, Tertiary education, Cancer, Hypertension, Rural/remote area, Immunocompromised, Q80 vax reduces

										severity, Born overseas, Q69 doses received, Diabetes
Booster 2023 (Q63)	4996	2544	15.2641 8	0.0655 13	0.839 6	0.019 1	25	18		Q78 vax reduces risk, Q69 doses received, Q80 vax reduces severity, Age, Q86 take antivirals, Q85 seek antivirals, Any Q32 risk factor, Renting, Q79 vax prevents infection, Risk stacked, Healthcare worker, Female gender, Immunocompromised, Hypertension, Obesity, Rural/remote area, Non-English at home, Heart disease
Booster 2024 (Q66)	5066	969	4.94171 3	0.2023 59	0.832 2	0.018	25	25		Q78 vax reduces risk, Q69 doses received, Q85 seek antivirals, Q79 vax prevents infection, Renting, Risk stacked, Any Q32 risk factor, Q80 vax reduces severity, Healthcare worker, Female gender, Any chronic condition, Age,

										**Currently employed, Tertiary education, Non-English at home, Born overseas, Asthma/Respiratory, Heart disease, Rural/remote area, Obesity, Cancer, Q86 take antivirals, Diabetes, Hypertension, Immunocompromised**
**Intention: new booster (Q74)**	**4131**	**2504**	**11.5139 54**	**0.0868 51**	**0.854 4**	**0.018**	**23**	**18**	**Q78 vax reduces risk, Q80 vax reduces severity**	**Q86 take antivirals, Q79 vax prevents infection, Age, Q69 doses received, Q85 seek antivirals, Renting, Tertiary education, Currently employed, Hypertension, Obesity, Non-English at home, Healthcare worker, Rural/remote area, Cancer, Any chronic condition, Female gender, Risk stacked, Born overseas**

**Annual booster intention (Q77)**	**4204**	**2534**	**6.551286**	**0.152642**	**0.8542**	**0.0128**	**23**	**18**	**Q78 vax reduces risk, Q80 vax reduces severity**	**Q86 take antivirals, Q79 vax prevents infection, Age, Q69 doses received, Q85 seek antivirals, Renting, Currently employed, Healthcare worker, Tertiary education, Risk stacked, Obesity, Non-English at home, Diabetes, Hypertension, Rural/remote area, Born overseas, Heart disease, Asthma/Respiratory**
**Willingness: antivirals (Q35)**	**5141**	**3339**	**15.26418**	**0.065513**	**0.7704**	**0.0153**	**24**	**19**	**Q86 take antivirals**	**Q78 vax reduces risk, Q85 seek antivirals, Q80 vax reduces severity, Age, Any chronic condition, Q79 vax prevents infection, Healthcare worker, Any Q32 risk factor, Hypertension, Immunocompromised, Female gender, Currently employed, Rural/remote area, Heart disease,**

										Asthma/Respiratory, Obesity, Tertiary education, Cancer, Q69 doses received
**Seek antivirals from GP (Q85)**	**5141**	**3514**	**1.59985 9**	**0.6250 55**	**0.561 6**	**0.033**	**24**	**23**	**Q85 seek antivirals**	**Q86 take antivirals, Q78 vax reduces risk, Female gender, Diabetes, Hypertension, Immunocompromised, Non-English at home, Q69 doses received, Tertiary education, Obesity, Born overseas, Age, Currently employed, Healthcare worker, Risk stacked, Cancer, Any Q32 risk factor, Q80 vax reduces severity, Renting, Heart disease, Q79 vax prevents infection, Rural/remote area, Asthma/Respiratory**
**Take antivirals if eligible (Q86)**	**5141**	**3385**	**2.12095 1**	**0.4714 87**	**0.795 7**	**0.018 1**	**24**	**23**	**Q86 take antivirals**	**Q78 vax reduces risk, Q85 seek antivirals, Age, Q80 vax reduces severity, Any chronic condition, Risk stacked, Q79 vax prevents**

										infection, Currently employed, Healthcare worker, Any Q32 risk factor, Tertiary education, Non-English at home, Hypertension, Cancer, Obesity, Female gender, Asthma/Respiratory, Heart disease, Renting, Immunocompromised, Born overseas, Q69 doses received, Rural/remote area
**Belief: vax reduces risk (Q78)**	**5141**	**3894**	**6.55128 6**	**0.1526 42**	**0.778 7**	**0.023 6**	**22**	**18**	**Q78 vax reduces risk, Q79 vax prevents infection, Q80 vax reduces severity**	**Q86 take antivirals, Q85 seek antivirals, Tertiary education, Q69 doses received, Risk stacked, Diabetes, Female gender, Any chronic condition, Born overseas, Renting, Obesity, Immunocompromised, Healthcare worker, Asthma/Respiratory, Cancer, Heart disease, Currently employed, Rural/remote area**

**Belief: vax prevents infection (Q79)**	**5141**	**1422**	**20.2358 96**	**0.0494 17**	**0.673 2**	**0.042**	**22**	**18**	**Q78 vax reduces risk, Q79 vax prevents infection, Q80 vax reduces severity**	**Q85 seek antivirals, Age, Female gender, Any Q32 risk factor, Non-English at home, Q86 take antivirals, Currently employed, Q69 doses received, Tertiary education, Any chronic condition, Hypertension, Healthcare worker, Diabetes, Born overseas, Immunocompromised, Renting, Obesity, Cancer**
**Belief: vax reduces severity (Q80)**	**5141**	**4092**	**8.68511 4**	**0.1151 4**	**0.774 3**	**0.014 2**	**22**	**18**	**Q78 vax reduces risk, Q79 vax prevents infection, Q80 vax reduces severity**	**Q86 take antivirals, Q69 doses received, Q85 seek antivirals, Tertiary education, Healthcare worker, Renting, Rural/remote area, Diabetes, Female gender, Born overseas, Asthma/Respiratory, Currently employed, Risk stacked, Hypertension, Heart disease, Obesity, Any**

										Q32 risk factor, Non-English at home
Boosters important (Q89)	4193	2800	4.941713	0.202359	0.8714	0.0204	23	20	Q78 vax reduces risk, Q80 vax reduces severity	Q86 take antivirals, Q79 vax prevents infection, Age, Tertiary education, Q69 doses received, Healthcare worker, Q85 seek antivirals, Risk stacked, Currently employed, Born overseas, Any chronic condition, Obesity, Non-English at home, Rural/remote area, Immunocompromised, Renting, Female gender, Hypertension, Any Q32 risk factor, Asthma/Respiratory

Supplementary Table S4

Model Fit Statistics

AUC = Area Under ROC Curve; AIC = Akaike Information Criterion; Pseudo-R^2 = McFadden R^2

Outcome	Model	N	AUC	AIC	Pseudo-R^2
Antiviral uptake (Q33)	Model 2 (Extended)	2562	0.7594	1916.61	0.1327
Antiviral uptake (Q33)	Model 3 (Full+LOO COM-B)	2562	0.7723	1888.21	0.1476
Booster 2023 (Q63)	Model 2 (Extended)	4996	0.7031	6308.54	0.0924
Booster 2023 (Q63)	Model 3 (Full+LOO COM-B)	4996	0.7462	5994.93	0.1383
Booster 2024 (Q66)	Model 2 (Extended)	5066	0.728	4455.45	0.1039
Booster 2024 (Q66)	Model 3 (Full+LOO COM-B)	5066	0.8281	3820.89	0.233
Intention: new booster (Q74)	Model 2 (Extended)	4131	0.6959	5091.83	0.0851
Intention: new booster (Q74)	Model 3 (Full+LOO COM-B)	4131	0.7571	4734.8	0.1503
Annual booster intention (Q77)	Model 2 (Extended)	4204	0.6909	5217.17	0.0807
Annual booster intention (Q77)	Model 3 (Full+LOO COM-B)	4204	0.7549	4842.21	0.1478
Willingness: antivirals (Q35)	Model 2 (Extended)	5141	0.6462	6366.7	0.0477
Willingness: antivirals (Q35)	Model 3 (Full+LOO COM-B)	5141	0.7409	5826	0.1295
Seek antivirals from GP (Q85)	Model 2 (Extended)	5141	0.5516	6399.69	0.0066
Seek antivirals from GP (Q85)	Model 3 (Full+LOO COM-B)	5141	0.5593	6397.96	0.0075
Take antivirals if eligible (Q86)	Model 2 (Extended)	5141	0.664	6227.02	0.0604
Take antivirals if eligible (Q86)	Model 3 (Full+LOO COM-B)	5141	0.7263	5866.85	0.1156
Belief: vax reduces risk (Q78)	Model 2 (Extended)	5141	0.6284	5529.73	0.0335
Belief: vax reduces risk (Q78)	Model 3 (Full+LOO COM-B)	5141	0.6951	5289.19	0.0764
Belief: vax prevents infection (Q79)	Model 2 (Extended)	5141	0.6374	5802.15	0.047
Belief: vax prevents infection (Q79)	Model 3 (Full+LOO COM-B)	5141	0.8958	3716.42	0.3917
Belief: vax reduces severity (Q80)	Model 2 (Extended)	5141	0.6252	5066.25	0.0308
Belief: vax reduces severity (Q80)	Model 3 (Full+LOO COM-B)	5141	0.6648	4944.86	0.0549
Boosters important (Q89)	Model 2 (Extended)	4193	0.6985	4900.24	0.0854
Boosters important (Q89)	Model 3 (Full+LOO COM-B)	4193	0.7656	4515.45	0.1583
